# Smart Aqueous Zinc Ion Battery: Operation Principles and Design Strategy

**DOI:** 10.1002/advs.202305201

**Published:** 2023-11-10

**Authors:** Xiaosheng Zhang, Caoer Jia, Jinyu Zhang, Linlin Zhang, Xuying Liu

**Affiliations:** ^1^ School of Materials Science and Engineering Zhengzhou Key Laboratory of Flexible Electronic Materials and Thin‐Film Technologies Zhengzhou University Zhengzhou 450001 P. R. China

**Keywords:** environmental adaptations, multifunctional integrated devices, operation principles, smart responses, zinc ion batteries

## Abstract

The zinc ion battery (ZIB) as a promising energy storage device has attracted great attention due to its high safety, low cost, high capacity, and the integrated smart functions. Herein, the working principles of smart responses, smart self‐charging, smart electrochromic as well as smart integration of the battery are summarized. Thus, this review enables to inspire researchers to design the novel functional battery devices for extending their application prospects. In addition, the critical factors associated with the performance of the smart ZIBs are comprehensively collected and discussed from the viewpoint of the intellectualized design. A profound understanding for correlating the design philosophy in cathode materials and electrolytes with the electrode interface is provided. To address the current challenging issues and the development of smart ZIB systems, a wide variety of emerging strategies regarding the integrated battery system is finally prospected.

## Graphical abstract

This review discusses the design of smart zinc ion batteries (ZIBs) in self‐charging, electrochromic, self‐healing, self‐protection, wide operating temperature range and their applications in different fields. The critical factors and design philosophy determining the function and performance of smart ZIBs are comprehensively analyzed from the intellectualized design, the main challenges and the development of smart zinc battery systems are discussed and prospected.

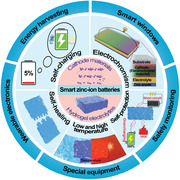

## 1 Introduction

Intelligent electronic devices such as wearable electronics, intelligent displays and electronic skins have been widely spread and used in modern society.^[^ ^1^, ^2^, ^3^, ^4^, ^5^ ^]^ Recently, it is in great demand for achieving smart electronic devices by functionally adjusting the chemical or physical properties and the process optimization including the single element or the complex hybrid systems.^[^ ^6^, ^7^, ^8^ ^]^ The battery as a power supply for integrated electronic devices is a very promising electrochemical energy storage system, it also puts forward more advanced intelligent requirements for battery systems in the future development of intelligent equipment.^[^ ^9^, ^10^ ^]^ Under the background of carbon neutralization, concerns about the unsustainability of fossil fuels and greenhouse gas emissions necessitate the development of new self‐charging rechargeable batteries that harvest clean energy.^[^ ^11^, ^12^, ^13^ ^]^ From the perspective of battery safety, it is necessary to consider the safety accidents caused by short circuit, heating, damage and other factors.^[^ ^14^, ^15^, ^16^ ^]^ In addition, battery failure at high or low temperatures is also an obstacle to the realization of advanced smart batteries.^[^ ^17^, ^18^ ^]^ This forced the development of an adaptive and self‐protective battery system to avoid battery failure.

Currently, a wide variety of lithium‐ion batteries with stimulus‐response have been developed to accommodate the external environment changes.^[^ ^19^, ^20^ ^]^ However, the limited application scenarios and the high‐cost of lithium sources as well as the high flammability of organic electrolytes still retards their applications.^[^ ^21^, ^22^, ^23^ ^]^ Consequently, according to the availability of the aforementioned responsive lithium battery, it is desirable to explore the novel and safe energy storage system. Various alkali metal ion batteries (Na^+^, K^+^) and multivalent ion batteries (Zn^2+^, Mg^2+^, Ca^2+^, Al^3+^,etc.) have been extensively developed. Zinc ion battery (ZIB) as one of the promising candidates in next‐generation battery systems has attracted much attention due to its high theoretical capacity (820 mAh g^−1^ and 5854 mAh cm^−3^), low redox potential (−0.763 V vs. a standard hydrogen electrode (SHE)), high safety, and abundant zinc resources.^[^ ^24^, ^25^ ^]^ In particular, the aqueous electrolyte of ZIB has low toxicity to the human body and favorable compatibility to the environment, which is suitable to be used as advanced energy in intelligent electronic devices.^[^ ^26^, ^27^ ^]^ However, there are still many challenges in ZIBs, such as the formation of zinc dendrites and the dissolution of cathode materials owing to their complexity and compatibility.^[^ ^28^, ^29^, ^30^ ^]^ Apart from the traditional design of electrodes, the design of smart materials has also utilized in ZIBs. The ingenious design of intelligent ZIBs utilizes functional electrodes or functional electrolytes to integrate functions such as energy harvesting and self‐protection into zinc batteries. For example, the novel charging patterns such as the self‐charging system utilizing air/photo are also reported in ZIBs, realizing clean energy harvesting and application in the independent grid charging system.^[^ ^31^ ^]^ In addition, the functional hydrogel electrolytes are fabricated to replace the traditional aqueous electrolytes for achieving smart properties of ZIBs including the self‐healing and thermal stimulus‐responsive characteristics (**Table** **1**).^[^ ^32^, ^33^ ^]^

**Table 1:** Comparison of properties of different alkali metals and multivalent metal ion batteries.^[^ ^204^, ^205^, ^206^ ^]^

Metal‐ion Battery	Ionicradius[Å]	Cost of metal anode [USD kg^−1^]	Charge density [C mm^−3^]	Theoretical volume specific capacity [mAh cm^−3^]	Electrolyte Type	Ionic conductivity [S cm^−1^]
Zn	0.75	2.2	112	5854	aqueous electrolytes	≈1‐10
Mg	0.72	2.2	120	3834		
Al	0.53	1.9	364	8046		
Li	0.76	19.2	52	2061	organic electrolytes	≈10^−3^
Na	1.02	3.1	24	1129		
K	1.38	13.1	11	610		

**Figure 1:**
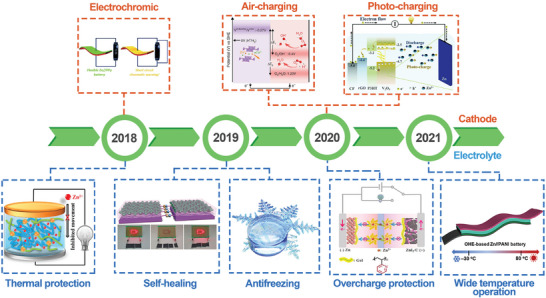
The brief development of the smart ZIBs. From left to right: Reproduced with permission.^[^ ^34^ ^]^ Copyright 2018, Elsevier. Reproduced with permission.^[^ ^35^ ^]^ Copyright 2018, Royal Society of Chemistry. Reproduced with permission.^[^ ^36^ ^]^ Copyright 2019, Wiley‐VCH. Reproduced with permission.^[^ ^37^ ^]^ Copyright 2019, Royal Society of Chemistry. Reproduced with permission.^[^ ^31^ ^]^ Copyright 2020, Springer Nature. Reproduced with permission.^[^ ^38^ ^]^ Copyright 2020, Wiley‐VCH. Reproduced with permission.^[^ ^39^ ^]^ Copyright 2020, Royal Society of Chemistry. Reproduced with permission.^[^ ^40^ ^]^ Copyright 2021, American Chemical Society.

In spite of wide investigations focusing on the design and integration of the smart battery, there is still no systematic and comprehensive review to correlate the sensitive structures with the energy storage performance. Herein, this review first summarizes the preparation methods and principles of realizing smart ZIBs and then focuses on the development of the smart ZIBs, including the design of the functional cathode materials and the intelligent hydrogel electrolytes. In addition, the corresponding reaction mechanism and the design strategies of the various intelligent materials in smart battery systems are also highlighted. Besides, the development route of the smart ZIBs and their application fields was proposed and summarized in **Figure** **1** for clearly exhibiting the landmark events. Finally, we present the challenges and future development of smart materials and their application in the ZIBs system, thereby providing new insights for smart devices.

## 2 The Smart Response Principles of ZIBs

### 2.1 Energy Harvesting and Utilization

**Figure 2:**
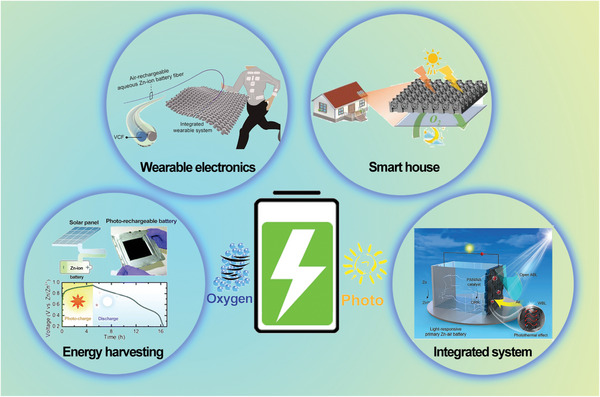
Application scenarios of self‐charging ZIBs. Reproduced with permission.^[^ ^39^ ^]^ Copyright 2020, Royal Society of Chemistry. Reproduced with permission.^[^ ^44^ ^]^ Copyright 2021, Royal Society of Chemistry. Reproduced with permission.^[^ ^45^ ^]^ Reproduced with permission.^[^ ^46^ ^]^ Copyright 2021, Wiley‐VCH.

The development of clean renewable energy is desirable to solve the unsustainability of fossil fuels and discontinuity of the wind energy and solar energy, as well as other negative impacts on the environment in modern society. Renewable energy sources such as power grids play an important role in commercial batteries in global energy supply.^[^ ^41^, ^42^, ^43^ ^]^ However, the electrical grid is not available in harsh environments or special areas (ocean and space) and is also difficult to provide a reliable energy supply. In addition, integrated energy harvesting devices and energy storage devices also increase costs and device complexity. Therefore, it is significant to construct self‐charging power systems to simplify the device configuration and provide the continuous energy supply at a certain solution. Inspired by the solar charging system, the self‐charging electrical device could be achieved through the natural environment. The chemical redox reaction as a type of traditional energy conversion is the available energy source and could be utilized to achieve self‐charging behavior. Moreover, the chemical redox based on the oxygen in the air also widely served as the active material in the metal‐air battery. As a consequence, the self‐charging property is also desirable for the energy storage supply in the smart device. The self‐charging batteries through air or sunlight effectively overcome the above problems, which provide a feasible way for off‐grid power demand such as wearable electronic devices or the energy supply for remote rural areas (**Figure** **2**). In consideration of safety, the aqueous ZIB with the self‐charging behavior also plays an important role in achieving the new‐type energy storage device and integrated smart battery system.

### 2.2 Stimulus Response

**Figure 3:**
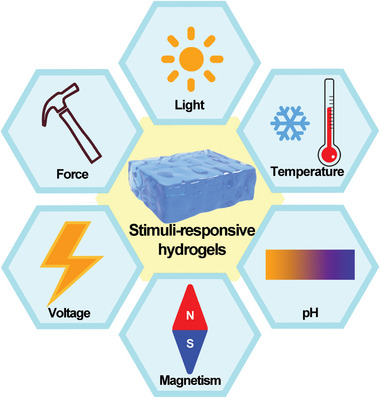
The stimulus‐responsive materials correspond to the external experimental changes including the voltage, force, light, temperature, pH, and magnetism.

**Figure 4:**
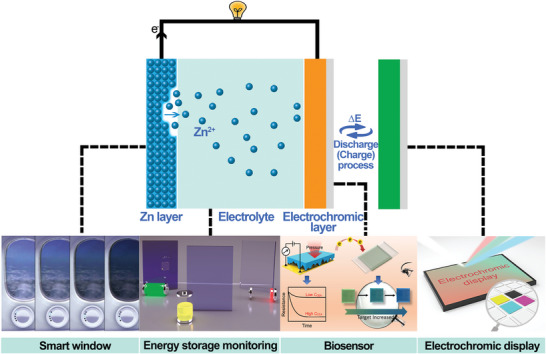
Principle and applications of the electrochromic materials such as the smart window, energy storage device, biosensor, and electrochromic display. From left to right: Boeing aircraft produced by SmartTintW. Reproduced with permission.^[^ ^56^ ^]^ Copyright 2016, American Chemical Society. Reproduced with permission.^[^ ^57^ ^]^ Copyright 2021, American Chemical Society. Reproduced with permission.^[^ ^55^ ^]^ Copyright 2022, American Chemical Society.

Materials with the “intelligent” behavior, that is, when the external environment such as the temperature, voltage, magnetism, force, light, or pH changes will make the corresponding transformation by changing the molecular structure or the chain skeleton distortion to adapt the external environmental changes (**Figure** **3**). However, materials are generally considered to be isolated and static, which can only achieve some low‐level functions without the ability to respond intelligently. To solve these issues, various kinds of stimuli‐responsive materials with different functional properties are integrated into hydrogels, nanomaterials, carbon nanotubes, and polymers and have been utilized in different fields such as drug delivery, tissue engineering, smart energy storage, soft robotics, optoelectronics, food science, etc.^[^ ^47^, ^48^, ^49^, ^50^ ^]^ For example, smart responsive hydrogel materials are used as drug delivery systems, wound dressings, biosensors and tissue substitutes in the field of biomedicine. The responsive nano‐protein particles are utilized to produce food and drugs, and the responsive liquid crystal polymers are used in the fields of soft robots, photonics and optoelectronics. Besides, the smart hydrogel materials serve as charge transfer carriers in the functional electrolytes of the intelligent energy storage. The preparation of the smart materials is necessary for achieving the self‐adapting property in the smart response fields and their further application in the smart responsive devices. The smart responsive materials were widely distributed in the pH, heat, and voltage in the ZIB system.^[^ ^51^ ^]^ They could effectively and intuitively monitor and avoid safety problems such as overcharging or overheating at a working battery system. In addition, the introduction of electrochromic smart materials is can also be very convenient to detect the change of the state of the ZIB system. The application fields of smart electrochromic devices are briefly distributed in the smart window, energy storage monitoring, sensor, and electrochromic display, etc. (**Figure** **4**).^[^ ^52^, ^53^, ^54^, ^55^ ^]^ The design of the smart response materials also plays an important role in achieving the smart devices with different external stimuli, and further are integrated into the energy storage and conversion system for sustainable development.

### 2.3 Self‐Healing

**Figure 5:**
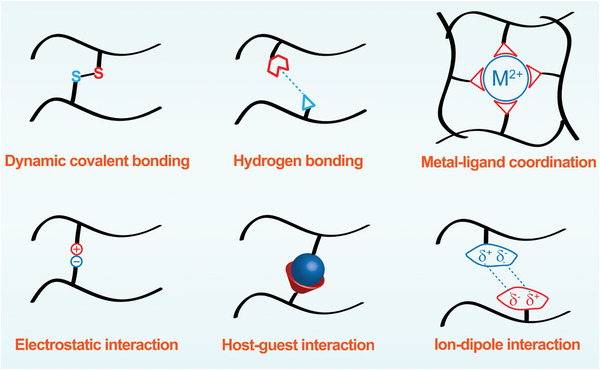
Self‐healing materials based on the physical/chemical reaction mechanisms, including the covalent bonding, hydrogen bonding, metal‐ligand coordination, electrostatic interaction, host‐guest interaction, and ion‐dipole interaction.

For the ZIB as an energy supply device, a certain degree of fatigue damage will inevitably occur during the practical application of the materials, which could shorten their service lifespan or lead to safety hazards to some extent, especially under special working conditions. It is desirable for constructing the new‐type materials to withstand the destruction derived from the external factors and avoid secondary disasters such as the short circuit of the working battery, especially with the flammable organic electrolyte. Inspired by the concept of the smart response, the self‐healing materials with the self‐repair management have been widely developed recently. To prolong the lifespan of the devices, abundant efforts have been devoted to developing smart materials with the self‐healing property. Self‐healing materials can be divided into active self‐healing materials and non‐active self‐healing materials. The active self‐healing materials can realize the self‐healing process without the triggering of external stimuli (similar to the wound healing). The non‐active self‐healing materials could realize the healing construction based on external stimuli, such as light, heat, pH, etc. As illustrated in **Figure** **5**, there are several strategies to achieve the self‐healing property in aqueous ZIBs, including dynamic covalent bonding, metal‐ligand coordination, hydrogen bonding, electrostatic interaction, host‐guest interaction and ion‐dipole interaction.^[^ ^58^, ^59^, ^60^, ^61^, ^62^, ^63^ ^]^ The design of the heal‐healing materials is utilized to achieve the normal working under different working conditions by self‐repairing the internal structure of the material through external stimulation or response. It is desirable for achieving self‐healing ZIB and provides an uninterrupted energy supply. Therefore, self‐healing materials have been widely used in electronic devices such as the smart energy storage devices, electronic skin, artificial muscles, and sensors.^[^ ^64^, ^65^, ^66^ ^]^

### 2.4 Smart Integrated Device

Modern electronic devices have been upgraded from single‐function isolated devices to smart integrated interactive devices, which greatly improves the user experience. Meanwhile, it also promotes the development of modern electronic devices, such as integration with flexible textiles, electronic skin and wearable electronics for achieving faster, more convenient and direct information acceptance and processing.^[^ ^67^, ^68^ ^]^ Multifunctional electronic devices can be integrated by multiple single‐function electronic devices, such as sensors integrated with energy harvesting devices to power the sensor.^[^ ^69^, ^70^ ^]^ However, poor compatibility and high cost of such multifunctional integrated devices is not an optimal choice in consideration of the complex configurations. To solve these issues, the design of the smart material with both energy storage and sensing functions would greatly simplify the device configuration, decrease the cost, and eliminate the compatibility concerns during the integrated process.^[^ ^71^ ^]^ For example, an all‐in‐one ZIB‐based pressure sensor is integrated with the functionality of a working battery and a common pressure sensor. The resistance of the device is changed by external pressure to convert mechanical signals into electrical signals for output, and at the same time, it can also be charged and discharged repeatedly as an energy supply device. This ZIB‐type sensor greatly solves the shortcomings of complex device integration, high cost, and large volume. As a result, the smart integration of a single device with multifunctional materials solves the above issues, which is also a promising direction for smart electronic devices.

## 3 The Multifunctional Design of Cathode Materials in Smart ZIBs

Cathode material as the important component in aqueous ZIBs determinates the working voltage, specific capacity, and cycling stability by the insertion/extraction of zinc ions during the charge/discharge process. In general, the requirements of cathode materials in the aqueous ZIBs with high performance are summarized as follows: 1) Structural stability for long‐term cycling life. 2) Abundant active sites for Zn^2+^ intercalation/de‐intercalation with fast kinetic reaction. 3) Appropriate operating voltage. 4) Low cost and environmental suitability. 5) High energy density and power density.

Currently, there are various electrode materials utilized in ZIBs, including vanadium‐based materials, manganese‐based materials, Prussian blue analogs, organic materials and other composites.^[^ ^72^, ^73^ ^]^ As a classic electrode material for zinc ion storage, manganese oxides are the first mentioned cathode materials and studied in ZIBs. There are several different reaction mechanisms of the manganese oxides (α‐MnO_2_, β‐MnO_2_, γ‐MnO_2_ and other manganese oxides).^[^ ^24^, ^74^ ^]^ Among which, the reversible zinc ion insertion/de‐insertion mechanism is widely accepted and developed, which involves a reversible phase transition between α‐MnO_2_ and spinel ZnMnO_4_.^[^ ^75^, ^76^ ^]^ However, the capacity decay derived from the irreversible Jahn‐Teller effect in electrochemical performance still limits its further application despite the high voltage platform.^[^ ^77^, ^78^ ^]^ For vanadium‐based materials, the diverse oxidation states and chemical properties endow the ZIB with high capacity and good rate performance.^[^ ^79^ ^]^ As a typical intercalation oxide cathode electrode, the reversible insertion and de‐insertion of Zn^2+^ is accompanied a certain amount of H^+^ and H_2_O in charging/discharging process.^[^ ^80^ ^]^ The layered vanadium‐based materials with large inter‐lamellar spacing are beneficial for achieving the high rate performance in comparison with manganese oxides materials. However, the dissolution of the vanadium in aqueous electrolyte is urgent to resolve. For Prussian blue analogs cathode, its large interstitial sites and special tunnels allow the reversible insertion/extraction of active diverse ions including Zn ions.^[^ ^81^ ^]^ The relatively low specific capacity and optional variety of Prussian blue analogs still limit its application. Different from the common inorganic compound electrode, the energy storage capability of organic cathodes such as conductive polymer (polyaniline) and quinone‐based material is dependent on the ion coordination effect between negatively charged atoms on the polymer chain or the functional groups.^[^ ^82^, ^83^ ^]^ The complex preparation process of organic materials is also not beneficial for the practical application. The layered structure with large spacing and a variety of chemical valence states as the important components promise the reversible intercalation/de‐intercalation of zinc ions to obtain the high specific capacity in an electrochemical process. To solve the aforementioned issues, a series of strategies were developed to improve the structure stability of active electrode materials by pre‐intercalating cation, structural water, and active defect.^[^ ^84^ ^]^

On the basis of considering the chemical reversibility and stability, the functional design of electrode material also plays an important role in achieving the smart battery system. And this issue would determine the application of smart energy storage devices in wearable electronic devices or other intelligent fields in future. A kind of cathode materials with special chemical or physical properties are explored and utilized in aqueous batteries, which provide potential possibilities for intelligent devices. In this section, we will summarize the smart cathode materials and their application in aqueous ZIBs and provide the potential design strategies for the smart electrode materials.

### 3.1 Cathode Materials with Energy Harvesting Function

As the reversible secondary battery system, it has been widely utilized in electronic devices such as mobile phones and electric vehicles because of its convenience and portability. However, the vast majority of electronic devices are recharged by the power grids at special charging stations, which is not available for the power supplement without grids. As a consequence, electronic devices without power supplies are unable to continuously work and affect our normal life to some extent, especially in the harsh environment or remote areas. Although abundant efforts have devoted to collecting external energy patterns (such as solar energy, mechanical energy, or thermal energy) to supply the electronic devices, the complex integration and conversion of these energy devices and their continuous property at all‐weather condition states still retard their widespread application in the present situation. Therefore, it is desirable to explore novel and simple charging patterns such as self‐charging mode to simplify this energy supply mode for achieving long‐term cycling properties in the smart battery system.^[^ ^85^, ^86^, ^87^ ^]^

#### 3.1.1 Air‐Charging Cathode Materials

Oxygen as an extremely abundant substance in the air apart from could release energy through the chemical redox reaction, such as burning and metabolism. Oxygen as the chemically stored energy is an available energy source and could be converted into electronic energy through a redox reaction. Inspired by this, the development of self‐charging aqueous ZIBs system would effectively resolve the above‐mentioned continuous energy supply and universality in all‐working conditions. For example, vanadium oxides as the common cathodes are easily oxidized by oxygen in the ZIB open air atmosphere when the vanadium element is in the reduced state (low chemical valence).^[^ ^88^ ^]^ The vanadium oxides were reduced with the insertion of Zn^2+^ during the discharging process, corresponding to the valence change of vanadium from V^5+^ to V^4+^ and V^3+^, respectively. The voltage of the vanadate composite at discharged state is much lower than the oxygen in the open air in the three‐electrode test system. It would result in the potential difference in comparison with the standard electrode potential of oxygen. Therefore, according to the relationship between thermodynamic function and battery voltage, the vanadate with low chemical valence could be oxidized in theory. During this oxidization process, oxygen was stored into the cathode materials in the aqueous ZIBs. The chemical oxidization of vanadium with the assistance of oxygen is similar to the traditional charging process, contributing to the smart charging mode without the external power supply. This novel phenomenon is defined as smart self‐charging behavior. It provides a new strategy for collecting energy through the timely charging mode.

**Figure 6:**
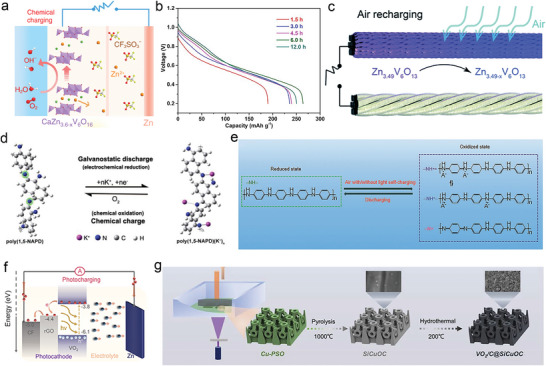
Self‐charging ZIBs. a) Working mechanism of chemically self‐charging ZIBs during a chemical charging process. Reproduced with permission.^[^ ^31^ ^]^ Copyright 2020, Springer Nature. b) Galvanostatic discharging profiles of the ssZIBs at 0.2 A g^−1^ after the oxidation of the VO_2_ cathode for different times. Reproduced with permission.^[^ ^89^ ^]^ Copyright 2021, Wiley‐VCH. c) Schematic to the VCF/Zn battery fiber during an air‐recharging process. Reproduced with permission.^[^ ^44^ ^]^ Copyright 2021, Royal Society of Chemistry. d) Self‐charging schematic diagram of poly(1,5‐NAPD)//Zn cell. Reproduced with permission.^[^ ^90^ ^]^ Copyright 2021, American Chemical Society. e) Proposed redox process at the cathode for different charging modes. Reproduced with permission.^[^ ^46^ ^]^ Copyright 2021, Wiley‐VCH. f) Schematic illustration of the proposed photocharging mechanism of VO_2_‐rGO photo‐ZIBs. Reproduced with permission.^[^ ^92^ ^]^ Copyright 2021, Wiley‐VCH. g) Schematic illustration of the LTSs preparation process. Reproduced with permission.^[^ ^45^ ^]^ Copyright 2023, Wiley‐VCH.

For example, Niu et al. has first reported the self‐charging battery with the CaV_3_O_8_ cathode in aqueous Zn(CF_3_SO_3_)_2_ electrolyte.^[^ ^31^ ^]^ Owing to the difference in redox potential between oxygen and the discharged product of CaZn_3.6_VO at discharging state, there is a spontaneous electron transfer between oxygen and CaZn_3.6_VO accompanied with the redox reactions (**Figure** **6**a). The oxidation of vanadium could be realized, and the self‐charging process would take place with the extraction of Zn^2+^ from the layered structure to balance the charge without any external power supply at the same time. It is a similar power charging behavior with a reversible process. In addition, based on the oxidation of vanadium element in an open air environment, another self‐charging all‐solid‐state ZIB (SS‐ZIB) was successfully fabricated using vanadium dioxide (VO_2_) as the cathode and polyacrylamide (PAM)‐chitin nanofiber (ChNF) hydrogel as the electrolyte (Figure 6b).^[^ ^89^ ^]^ Based on these redox action of vanadate oxides (Figure 6c), some freestanding fiber cathodes or the multi‐dimensional structure electrodes were also prepared in the self‐charging ZIB system.

Apart from the inorganic materials as cathodes in ZIBs, organic materials with diverse and designable structures are also utilized in aqueous zinc battery. During the charge/discharge process, the rearrangement of chemical bonds in organic materials is susceptible to redox reactions and accompanied by special self‐charging behavior. Wan et al. constructed a zinc‐organic battery with the self‐charging property.^[^ ^90^ ^]^ The poly (1,5‐naphthalenediamine) was the cathode electrode in 6 M KOH/0.2 M Zn(CH_3_COO)_2_ electrolyte. Based on the conversion of the C═N/C─N bond, the reversible zinc storage achieved a high capacity of 188.9 mAh g^−1^. When the battery was discharged to a low voltage, the fully discharged product poly(1,5‐NAPD) (K^+^)n with weak binding energy was easily oxidized by the oxygen (Figure 6d). The design of such organic cathode materials broadens the application of ZIBs and would be beneficial for achieving a fast self‐charging process. However, the relatively low working voltage and capacity are still a challenge for assembling the high‐energy battery system. Moreover, the self‐charging behavior will proceed in an irreversible direction during the repeated self‐charging process. With the continuous self‐charging process, the formation of by‐product alkaline zinc salt massively accumulates on the surface of the active electrode, which seriously affects the intercalation of Zn^2+^ into the electrode during the power charging process. Therefore, the rational regulation of the oxidization time and the condition of electrolytes in the charging/discharging process plays an important role in realizing the reversible electrochemical performance in ZIBs with self‐charging functions. The flexible and environmentally friendly solid‐state ZIBs system is very suitable for wearable electronic devices, and its self‐charging function is easily charged in any scenario.

To extend the self‐charging working process, the ZIB with multiple modes was also carried out to avoid the drawbacks of a single battery system. Inspired by this phenomenon, a multi‐mode switching smart zinc battery system via “All‐in‐One” polymer cathodes was successfully fabricated. The active cathode (PANINA/CC@WBL@ABL) in the battery was composed of PANINA grown on carbon cloth (CC), a waterproof and breathable layer (WBL), and a transparent air barrier layer (ABL) to achieve three different working modes.^[^ ^46^ ^]^ Among which, the PANINA was used as the redox‐active species and oxygen reduction electrocatalysts with a photothermal‐responsive (Figure 6e). 1) It is a typical ZIBs mode with a capacity of 430 mAh g^−1^, in which ABL is turned off; 2) The ABL was turned on after the battery was fully discharged. The reduced PANINA reacted with oxygen in the air, which was equivalent to the charging process of the battery; 3) The ABL was opened, and the battery was switched to Zn‐air battery in the fully discharged state. The photo‐thermal effect of PANINA significantly improved the electrochemical performance and self‐charging efficiency in the presence of light.

#### 3.1.2 Photo‐Charging Cathode Materials

In addition to oxygen, sunlight is also considered to be an inexhaustible renewable clean energy. The harvest and utilization of clean energy is an important way to solve the pressing need of energy saving and emission reduction. The integrated system of solar cells and rechargeable batteries with complex configurations increases energy loss.^[^ ^91^ ^]^ The design of bi‐functional photo‐active materials with energy harvesting and storage solved these problems. Vanadium pentoxide (V_2_O_5_) nanofibers mixed with poly(3‐hexylthiophene‐2,5‐diyl) (P3HT) were used as photo‐recharging active materials, which both realized the bi‐functions of solar energy harvesting and charge storage.^[^ ^39^ ^]^ The energy levels of P3HT and rGO allowed the transport of photo‐excited electrons from V_2_O_5_ nanofibers to the current collector, and the unpaired photo‐induced holes were blocked by P2HT and accumulated in the photo‐active material. The capacity of ZIB with the photo‐active material as the cathode increases from 190 to 370 mAh g^−1^ in illuminated condition with ≈1.2% photo‐conversion efficiencies. The 100 cm^2^ large‐scale pouch ZIBs also achieve a long‐term cycle of photo‐charging/constant‐current discharging process, demonstrating its potential off‐grid charging applications. To reduce the cost of the integrated devices, the reduced graphene oxide with the photoelectric conversion function was introduced into the energy storage and conversion system.^[^ ^92^ ^]^ The photoactive positive electrode prepared by mixing vanadium dioxide and rGO offers the necessary charge separation and storage for photo‐charging (Figure 6f).

Apart from the vanadium oxides, MoS_2_ is also used as a photo‐active material in photo‐rechargeable ZIBs by generating photo‐excited electron‐hole pairs and acting as a carrier for storing zinc ions.^[^ ^93^ ^]^ In the integrated smart device, the capacity increases from 245 to 340 mAh g^−1^ at a light power of 12 mW cm^−2^ at 455 nm. The design of the binder‐free photo‐cathode material could further increase the photo‐electric conversion efficiency, which could effectively promote energy storage and conversion in integrated smart devices, especially for the response‐based battery system. The sufficient capacity and light utilization efficiency are the significant parameters for realizing the expected photo‐charging ZIB. However, the low specific surface area of the flat electrode reduces its light utilization efficiency, and the flat ZIB cannot be effectively illuminated after being packaged and wrapped. Therefore, a new class of 3D light‐trapping structures (LTSs) was proposed for the practical photo‐charging ZIBs.^[^ ^45^ ^]^ The large specific surface area exhibited a 400% photo response current density in comparison with the reported flat electrode and delivered 0.19 mWh cm^−2^ at 0.51 mW cm^−2^, which is attributed to the enhanced multiple internal reflections and the large surface area (Figure 6g). The simulated integration of photo‐charging ZIB into the roof for power supply based on the rigid SiCuOC possessed a high enough strength (over 9 Mpa) for guaranteeing its potential application. The photo‐charging smart ZIB could provide the continuous powered supply, which is a living example for simulating the actual environment of a dark environment.

As a consequence, self‐charging ZIBs easily charge using air or photo without external power grids. It greatly meets the application requirements of electronic equipment in special scenarios, especially in the next‐generation energy storage devices. The current challenges and corresponding design strategies of the self‐charging ZIBs are as follows:

1. The exposed environment for realizing the self‐charging process would lead to the irreversible electrolyte leakage or evaporation, causing the capacity decay and the poor cycling stability or safety hazards. The corresponding solution is to develop the new encapsulation materials and battery structure. For example, the selectively permeable packaging materials with inner hydrophobicity only allow air permeability and effectively prevent the loss of the liquid electrolyte.
2. The short self‐charging time is necessary for the practical application. The self‐charging time is mainly determined by the redox reaction process in the cathode electrode during the oxygen charging or the photo charging process. The corresponding solution is mainly the rapid charge transfer on the cathode electrode. a) The construction of the hierarchical porous structure of the electrode provides the large contact surface area, which contributes to the abundant physical space and active sites for the redox reaction. b) The content adjustment of external influencing factors is also desirable for improving the redox reaction process, such as the oxygen contents in the electrolytes, the duration time at exposure atmosphere, or the control of the photo power for the photo‐charging process.
3. The high energy density and availability of self‐charging ZIBs. High theoretical capacity and open circuit voltage determinate the energy density of the battery. To extend its practical application, the dual‐ion insertion or the organic/inorganic hybridized cathode materials will be the possible alternatives in future for the high energy density self‐charging battery.^[^ ^94^, ^95^ ^]^
4. The reaction mechanism of self‐charging ZIBs is not well understood. During the self‐charging process, the oxidization of the cathode is also accompanied with other side reactions, such as the solid electrolyte interlayer on the cathode. It would affect the cycling stability and the reproducibility of the self‐charging behavior. Therefore, the in‐situ or the real‐time detection of the cathode and electrolyte is also desirable, including the structural analysis and chemical environment analysis. In addition, the booming theoretical simulation calculations could also provide the essential insights into the self‐charging reaction mechanism.
5. The integration of the self‐charging ZIBs in wearable electronic devices or the micro‐integrated electronic textiles. The traditional coin‐type battery is not suitable for the wearable devices. Therefore, the variously selectable shape of ZIB is desirable for promising the energy supplying and the self‐charging process.

### 3.2 Cathode Materials with Electrochromic Function

Intelligence transforms the life experience. With the development of the intelligence system, the corresponding devices with the visual control in the human‐machine interface. Currently, the electronic devices operate at the visible region is desirable for detecting the real‐time working variation in the devices, especially in the fields of the smart electronic devices.^[^ ^96^ ^]^ The electrochromic energy storage devices have attracted great interest recently, especially in the fields of smart windows, electrochromic displays, and electronic skins.^[^ ^97^, ^98^, ^99^ ^]^ Furthermore, the optical properties such as the reflectance, transmittance, and absorptivity of the electrochromic materials would undergo a stable and reversible color change under the application of the electric field or the change of the external voltage, which is called electrochromism.^[^ ^35^, ^96^ ^]^ These color changes in the devices would reflect the important information of these devices and play an important role in warning or remaindering functions. Consequently, these electrochromic materials and energy storage devices can be integrated by utilizing the voltage change of the battery during the charging and discharging processes to achieve the diversified display or even the early warning function.^[^ ^100^ ^]^

**Figure 7:**
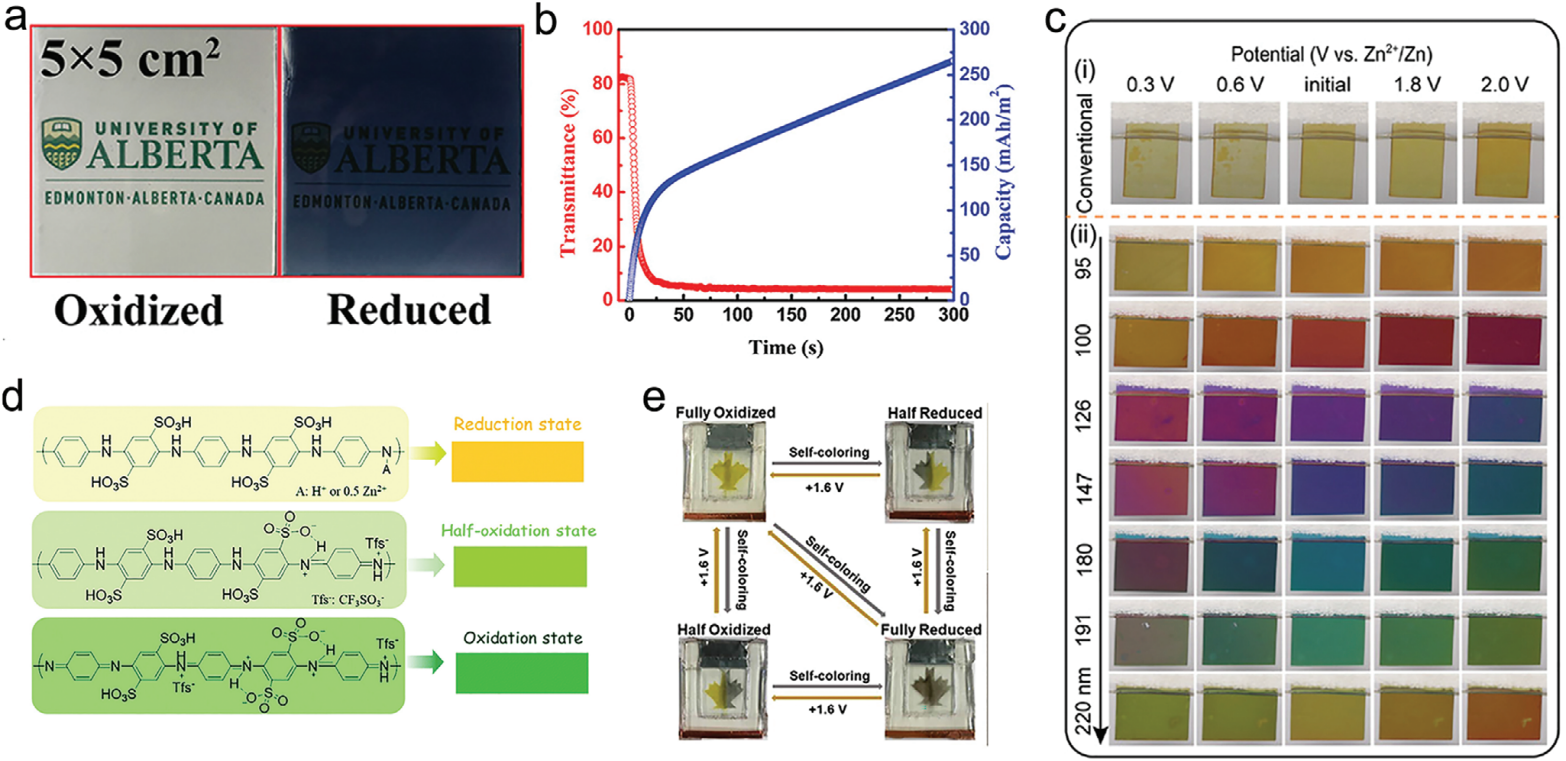
The electrochromic materials in ZIBs. a) Photographs of a 5 × 5 cm^2^ MTWO cathode before and after self‐coloring. b) In situ self‐coloring (discharge) process of the MTWO cathode. a and b) Reproduced with permission.^[[^ ^108^ ^]^ Copyright 2019, Wiley‐VCH. c) Optical images of i) conventional and ii) colorful electrochromic electrodes at different voltages (vs. Zn^2+^/Zn). Reproduced with permission.^[^ ^109^ ^]^ Copyright 2021, Wiley‐VCH. d) Molecular structure and electrochromic effect of self‐doped polyaniline in different oxidation states. Reproduced with permission.^[^ ^110^ ^]^ Copyright 2020, Royal Society of Chemistry. e) The functionalities of the electrochromic battery display were demonstrated with digital photographs. Reproduced with permission.^[^ ^111^ ^]^ Copyright 2019, Wiley‐VCH.

The battery as the energy storage and conversion device possesses an electrochromic function that can easily monitor residual capacity of the electronic devices through the variation of battery color. The electrochromic batteries can maintain only one constant color when the voltage is constant without the continuous energy consumption, which will be the optimal choice under the extreme conditions. The current collectors of the electrochromic material are usually transparent to observe the changes of color. There are two different electrochromic materials including inorganic and organic materials. The mechanism of inorganic electrochromic materials is the d‐level splitting of transition metal atoms through ion external insertion/extraction process.^[^ ^101^ ^]^ For example, WO_3_ as the first electrochromic material could realize the color change by injecting the electrons and the inserting metal ions (H, Li or Na). During the process, the corresponding valence state of tungsten in WO_3_ is reduced from hexavalent to pentavalent, and the color of WO_3_ changes from transparent to dark blue.^[^ ^102^ ^]^ Besides, MoO_3_, TiO_2_, NiO, V_2_O_5_ and other transition metal oxides with variable chemical valence are also utilized in the electrochromic fields due to their variable chemical valences in electrochemical process.^[^ ^103^, ^104^, ^105^ ^]^ For organic electrochromic materials, the reaction mechanism mainly includes small organic molecules (viologen and its derivatives) and the conductive polymers (such as polyaniline, polypyrrole, polythiophene).^[^ ^106^ ^]^ Their discoloration mechanism is mainly dependent on the direct electron transfer on the polymer chain or the functional groups on the branched chain with the ion doping or ion adsorption.^[^ ^107^ ^]^ For example, the electrochromic properties of polyaniline are determined by the dynamic ion doping/de‐doping process. The corresponding optical properties are controlled by the delocalization of p electrons in the polymer structure. These electrochromic materials also provide potential application in smart energy storage devices due to the color changes at an external voltage. The tungsten molybdenum oxide as the cathode material is utilized in the rechargeable aqueous zinc ion electrochromic battery (ZIEB).^[^ ^108^ ^]^ The cation vacancies could greatly improve the electrochemical performance of ZIEB by increasing the electrochemical activity of Zn‐ions. The smart color response occurs accompanied with the process of embedding Zn‐ions into the cathode, which is caused by the MTWO (self‐coloring) (**Figure** **7**a). The MTWO cathode displayed a strong optical contrast of ≈76% at 632.8 nm (Figure 7b) and a maximum optical contrast of 90% within only 14 s of self‐coloring time. During the self‐coloring process, the ZIEB could also deliver a high area specific capacity with 69% optical contrast. The integrated electrochromic performance and electrochemical performance based on the reconstruction of the electron structure could provide a new idea for the design of electrochromic batteries.

An electrochromic ZIB with manganese oxide as the cathode electrode exhibited the multiple distinct colors when operating at different potentials.^[^ ^109^ ^]^ Different from the conventional Mn_2_O_3_ electrode, the bilayer structures of Ti and Mn_2_O_3_ layers with modulated thicknesses possess a rich color gamut. The as‐assembled battery could present up to seven diverse colors by modulating the thickness of the manganese oxide layer in comparison with the only brown color of the conventional Mn_2_O_3_ (Figure 7c). At different voltages, the electrode could deliver violet at 2.0 V, crimson at 1.8 V, orange at 0.6 V, and coral at 0.3 V, respectively. As a consequence, the fabricated ZIB could present a series of colors at different voltage plateaus, which is beneficial for achieving the real‐time detection of the residual capacity. In addition, polyaniline with different oxidation or reduction states could also exhibit the different colors.^[^ ^110^ ^]^ During the storage of the cations in the electrochemical process, there are three oxidation states of polyaniline cathode: the full reduction state, the half‐oxidization state and the full oxidation state accompanied by the color switching from green to yellow (Figure 7d). As a result, the corresponding color of the battery with the modified polyaniline will also gradually change from light yellow to dark‐green at different voltages, which could demonstrate the intelligent feature of the energy storage state in a working battery (switching from a 100% full‐charged battery to low battery state). When the battery was re‐charged to a full charge state, the color of the battery could also recover to its initial state, which also reveals the reversible variable coloration feature in the battery system.

The visible color change in a working battery is realized under the operation of the external electronic field, which could unavoidably consume energy such as the electric energy. If there is a green method to trigger the color change without much energy consumption, it would be exciting. Therefore, it is desirable that the energy consumed in the electrochromic battery could be supplied during the discharging process or the other forms of energy could be converted into the driving force of the color change in a working battery through an energy recovery function. Li et al. have successfully synthesized the V_3_O_7_ colloidal particle with electrochromic function as ZIB cathode electrode for electrochromic display.^[^ ^111^ ^]^ When zinc ions were inserted or extracted, V_3_O_7_ accordingly switched between gray‐blue (coloring process) and yellow (bleaching process) with the response time of 10.4 and 28.6 s (the coloring efficiency was 20.6 cm^2^ C^−1^). During the electrochemical process, the electrochromic display with energy retrieval function switches between fully yellow, fully grayish‐blue, and half yellow‐half grayish‐blue (Figure 7e). The recovered energy could also power the LED for lighting 28 min, which further confirms that the electrochromic display in the coloring process achieved zero energy consumption, and recovered a moiety of energy to compensate for fading process energy consumption. Apart from the design of the single cathode materials, the integration of the battery system including the anode, the separator, the electrolyte or the external packing should also be taken into consideration.^[^ ^112^ ^]^ As a result, the design of these devices with the energy recovery function displays the novel working pattern in the visual smart energy storage devices.

## 4 The Multifunctional Design of Hydrogel Electrolyte in Smart ZIBs

Large‐scale, high‐safety and low‐cost energy storage equipment is regarded as the next‐generation substitute for meeting the great demand for new‐type electronic devices in comparison with the current commercial battery system. The leakage of liquid organic electrolytes and the induced flammable issues of the traditional lithium ion battery is still a challenge in wearable and implantable devices in the human body although the ionic conductivity is high.^[^ ^113^ ^]^ Therefore, the solid electrolyte has attracted abundant interest recently due to their high safety and ability to effectively prevent pollution or corrosion caused by electrolyte leakage.^[^ ^114^, ^115^ ^]^ Moreover, the strong mechanical properties of the all‐solid or quasi‐solid electrolyte could withstand the extreme pressure and external deformation, contributing to the cycling stability of the battery.^[^ ^116^, ^117^ ^]^ In addition, the solid electrolyte with the less free water can greatly avoid the occurrence of side reactions and the formation of zinc dendrites. Therefore, the solid electrolyte is a promising candidate for realizing the flexible wearable electronic devices. However, the corresponding slow migration of zinc ions in all‐solid electrolytes still leads to the low ionic conductivity of all‐solid flexible devices.^[^ ^118^, ^119^ ^]^ The lack of sufficient electrolyte wetting at the electrode/electrolyte interface causes the poor contact and the increased charge transfer resistance at the electrode interface, which hinders the kinetic reaction and the utilization of the active electrode and limits the further development of high‐performance all‐solid‐state ZIBs.^[^ ^120^, ^121^ ^]^ In consideration of the excellent electrochemical kinetic and environmental friendliness in a working battery system, the quasi‐state or all‐solid hydrogel electrolyte composed with the polymer skeleton and abundant functional groups is gradually explored. In addition, the diverse hydrophilic groups in the hydrogel polymer molecular chains lock a large number of water molecules into the voids inside the hydrogel network, contributing to the flexible and moist property in assembled devices. The cross‐linked 2D or 3D network would increase the strength of the hydrogel and change its elasticity and display the good mechanical property, which extend their application in flexible devices. Therefore, the content of free water, electrolyte salt, the external additive of solvents could effectively promote the electrochemical activity and reversibility and realize the high performance and good mechanical property in a flexible device based on the hydrogel electrolyte, quasi‐solid electrolyte, or the all‐solid electrolyte.

Traditional hydrogel materials are usually divided into natural polymer hydrogels and synthetic polymer hydrogels. Natural polymers (sodium alginate, collagen, fibrin, agarose, gelatin) possess many hydrophilic groups on the molecular chains (‐NH_2_, ‐OH, ‐COOH), which could serve as the physical crosslinking points for forming the gel electrolyte.^[^ ^122^, ^123^, ^124^, ^125^ ^]^ In addition, there are also synthetic polymer hydrogels based on the different polymer skeletons such as polyethylene glycol (PEG), polyacrylamide (PAM), or polyvinylalcohol (PVA), polyacrylicacid (PAA), sodium polyacrylate (PANa).^[^ ^126^, ^127^, ^128^, ^129^ ^]^ For example, the polyacrylamide as hydrogel skeleton was first utilized to engineer the high‐performance, waterproof, tailorable, and stretchable ZIBs. Then, the polyvinyl alcohol‐based hydrogel electrolyte with Zn(CF_3_SO_3_)_2_ as electrolyte salt was widely utilized in aqueous zinc battery due to its hydrophilic and film‐forming property. The assembled quasi‐solid‐state ZIB delivers the excellent electrochemical performance, high flexibility and high temperature stability. Besides, the water‐in‐salt hydrogel electrolytes were also developed to decrease the corrosion reaction between the free water and the zinc metal, such as the water‐in‐salt polyacrylamide electrolyte with 1 M Zn (TFSI)_2_ and 21 M LiTFSI.

In comparison with traditional polymers, functional hydrogels with smart features could be obtained by introducing additional functional groups or chemically changing the interface/surface properties.^[^ ^130^ ^]^ For example, the chemical and physical cross‐linked interaction or the weak interaction between molecular of the polymers could endow the hydrogel with self‐healing, self‐recovery, thermo‐responsive, pH‐responsive, and adaptability over a wide temperature range.^[^ ^112^, ^131^ ^]^ Besides, the hydrogel with the electrolyte salt could also provide the high ionic conductivity and good compatibility with the electrode, contributing to achieving the high‐performance flexible ZIB. Herein, we will discuss the design, preparation, functionalization, and the editability of the smart hydrogel in ZIBs (**Table** **2**).

**Table 2:** Summary of strategies and the electrochemical performance of ZIBs based on the hydrogel electrolytes with self‐healing function and temperature window.

Hydrogel electrolyte	Cathode	Capacity (mAh g^−1^)	Capacity retention	Self‐healing principle	Healing time	Working window	Ref.
PSBMA/ZnSO_4_	MnO_2_@ CNT	≈225.0 (1.5 A g^−1^)	600 cycles (1.5 A g^−1^)	electrostatic interaction	24 h	−20–25 ^o^C	[132]
Zn(CF_3_SO_3_)_2_/ chitosan /PAAM	PANI	≈210.0 (1.0 A g^−1^)	94.6% after 2000 cycles (3 A g^−1^)	reversible coordination and hydrogen bond	/	/	[133]
Cellulose/TEOS/ glycerol/ZnSO_4_	MnO_2_	277.3 (0.2 A g^−1^)	99.2% after 2000 cycles (3 A g^−1^)	hydrogen bond and Si─O─Si bond	2 h	−40–60 ^o^C	[134]
PVA/Zn(CF_3_SO_3_)_2_	PANI@ SWCNT	123.0 (0.1 A g^−1^)	97.1% after 1000 cycles (1 A g^−1^)	hydrogen bond	0.5 h	/	[36]
PVA/Zn(CH_3_COO)_2_/ Mn(CH_3_COO)_2_	VS_2_/CC	175.0 (0.2 A g^−1^)	70.3% after 40 cycles (0.2 A g^−1^)	hydrogen bond	0.5 h	/	[135]
PVA/borax/glycerol/ ZnSO_4_/MnSO_4_	rGO/ MnO_2_	242.5 (0.5 A g^−1^)	93.7% after 2000 cycles (1 A g^−1^)	/	/	−35–25 °C	[136]
PAAM/ZnSO_4_/LiCl	LiFePO_4_	106.0 (0.1 A g^−1^)	100.0% after 500 cycles (0.5 A g^−1^)	/	/	−20–25 °C	[137]
PAMPS/PAAm/ ZnCl_2_/NH_4_Cl/EG	PANI	207.7 (0.2 A g^−1^)	81.5% after 4000 cycles (5 A g^−1^)	/	/	−30–80 °C	[40]
PAM/glycerol/AN/ ZnSO_4_	V_2_O_5_	185.0 (5 A g^−1^)	88.0% after 10000 cycles (5 A g^−1^)	/	/	−20–60 °C	[138]

### 4.1 Self‐Healing Hydrogel Electrolytes

The design of self‐healing materials plays an important role in the stability, the performance, and the safety of working battery devices operated under extreme working conditions. When the flexible battery experiences damage or fatigue under an external force, the self‐healing mechanism would overcome the cracks or failure of the devices and prolong the lifetime and promise the strength. Therefore, the design of self‐healing features in the flexible devices is beneficial for achieving the smart intelligence in the practical applications.

Self‐healing hydrogels are defined as hydrogels capable of spontaneously bonding to their original shape and recovering mechanical properties after physical damage.^[^ ^139^ ^]^ Generally, there are two ways for hydrogel self‐healing: chemical cross‐linking and physical crosslinking. Physical cross‐linked self‐healing hydrogels realize spontaneous healing through covalent bonding, metal‐ligand coordination, hydrogen bonding, electrostatic interaction, host‐guest interaction and ion‐dipole interaction without external stimuli factors.^[^ ^140^, ^141^, ^142^, ^143^ ^]^ Chemical cross‐linked self‐healing hydrogels require external stimuli to realize the healing process, such as pH, and UV light or other chemical reactions.^[^ ^144^, ^145^ ^]^ To achieve the self‐healing effects in various situations, mixed chemical and physical interactions are induced into the battery system. The inevitable crush and deformation of the secondary batteries lead to battery damage in the long‐term cycle processes, which increases the probability of safety accidents. Self‐healing materials used in secondary batteries can avoid serious consequences caused by damage accumulation of batteries.

**Figure 8:**
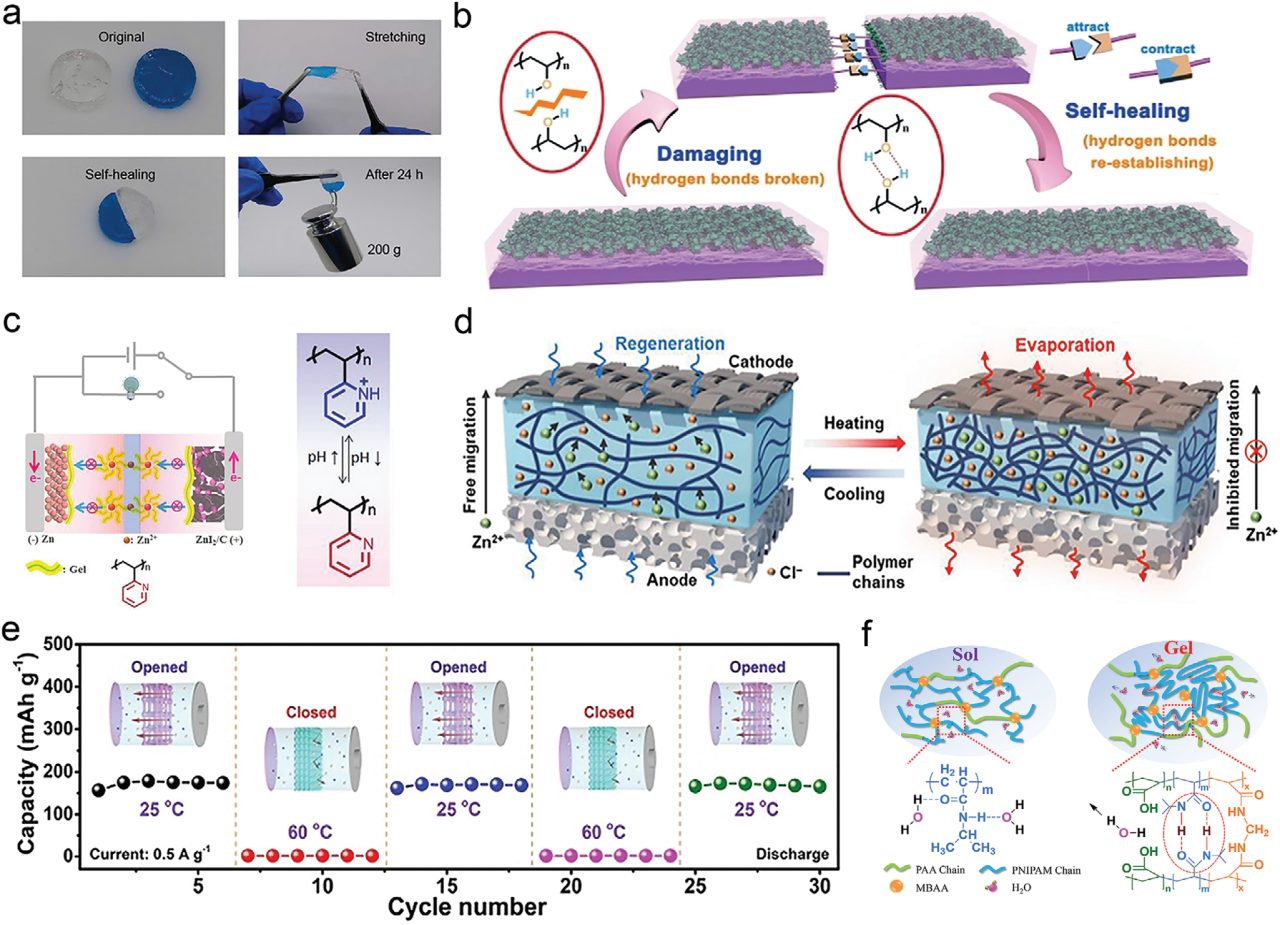
The smart hydrogel electrolytes in ZIBs. a) Self‐healing hydrogel electrolyte. Reproduced with permission.^[^ ^132^ ^]^ Copyright 2020, Wiley‐VCH. b) Self‐healing integrated all‐in‐one ZIBs. Reproduced with permission.^[^ ^36^ ^]^ Copyright 2019, Wiley‐VCH. c) Illustration of the self‐protection ZIABs. Reproduced with permission.^[^ ^38^ ^]^ Copyright 2020, Wiley‐VCH d) Working principle of the thermal self‐protective ZIBs based on hygroscopic hydrogel electrolyte. Reproduced with permission.^[^ ^153^ ^]^ Copyright 2020, Wiley‐VCH. e) The thermal‐responsive reversibility of the aqueous ZIBs. Reproduced with permission.^[^ ^152^ ^]^ Copyright 2020, Wiley‐VCH. f) Mechanism of the reversible sol‐gel transition of PNA copolymer. Reproduced with permission. Copyright 2018, Elsevier.

The self‐healing property of electrode or electrolyte or current carriers is the key factor to achieve the self‐healing battery devices. The electrostatic interaction of molecules is the key parameter to achieve the self‐healing property in the hydrogel electrolyte.^[^ ^132^ ^]^ The high water retention capacity and ion transport channels with abundant branches endow the electrolyte with the high ionic conductivity, which is beneficial for the rapid electrochemical process. In addition, the electrochemical interaction between charged groups and the hydrogen bonding between SO_3_^−^ groups and water molecules could facilitate the reconnection within the broken two‐part gel. Its reconstruction of the electrolyte interface could also maintain its original mechanical strength after standing for 24 h and sustaining the 200 g of mass (**Figure** **8**a). The excellent self‐healing performance of the hydrogel electrolyte is widely utilized in flexible aqueous battery systems. The supramolecular interaction also contributes to achieving self‐healing of hydrogel electrolytes in the high‐performance wearable ZIBs. Shu et al. designed a self‐healing chitosan chain/polyacrylamide hydrogel (Zn^2+^‐CS/PAAM) for high‐performance wearable ZIBs.^[^ ^133^ ^]^ Among which, the reversible coordination interaction between zinc ions and amino groups and the strong internal hydrogen bonds in aqueous electrolyte simultaneously promote the self‐healing behavior and overcome the possible physical damage in the wearable electronic devices. Furthermore, the abundant hydroxyl groups endow the hydrogel electrolyte with the excellent self‐healing properties and good mechanical properties with high elasticity and tensile strength. An all‐round hydrogel electrolyte was prepared by using cellulose‐containing cotton as hydrogel skeleton, tetraethyl orthosilicate, and glycerol as a cross linker and anti‐freezing agent, respectively.^[^ ^134^ ^]^ The addition of the glycerol could decrease the freezing point by retarding the evaporation of solvent water through the hydrogen bonds between the hydroxyl groups and water molecules, contributing to its application in low‐temperature environment. Without any external interaction, the repaired hydrogel electrolyte was reconnected without obvious cracks and could maintain a high self‐healing efficiency of 82.6%. It is ascribed to the reversible reform interaction of abundant hydrogen bonds within the hydrogel skeleton and Si─O─Si bonds.

The good hydrogen bond interaction between the polar functional groups of the hydrogel polymer skeletons plays a significant role in achieving the self‐healing property, especially for extending the practical application of aqueous zinc battery in smart wearable devices in future. Consequentially, the polymer with a large amount of hydrophilic functional groups is desirable for fabricating the self‐healing hydrogel electrolyte due to the formation of hydrogen bond interaction between the branches of the polymer skeleton. For example, the hydroxyl side groups on chain segments of the poly (vinyl alcohol)‐based (PVA) hydrogel could also achieve the self‐healing property once cut by an external force because of the spontaneous hydrogen bonding interaction when the repaired hydrogels connect together. A PVA‐based hydrogel (PVA/Zn(CF_3_SO_3_)_2_) was fabricated via a facile freeze/thaw approach.^[^ ^36^ ^]^ A large amount of PVA crystalline micro‐regions at low temperatures could serve as the cross‐linking agent during the formation PVA gel hydrogel and guarantee the 3D network with porous structure, providing the good ionic conductivity. When the PVA‐based electrolyte was damaged, the exposed uncrystallized PVA segments on the fracture surface could interact and form the hydrogen bonding and finally complete the self‐healing process (Figure 8b). Two parts of the damaged ZIB based on the hydrogel electrolyte could also normally only through the simple direct contact after being cut off and display the similar electrochemical performance. Besides, the good adhesion of the gel electrolyte with good flexibility is also beneficial for tailoring the shapes and patterns of the battery, demonstrating its wide application in stretchable and printable electronics.

Apart from hydrogel electrolyte with a single self‐healing function, the integrated electrode with the electrolyte is desirable for the full flexibility of the wearable devices, especially at the extreme condition. Therefore, the all‐in‐one type electrode with reversible self‐healing property is explored. With the flexible collector substrate, the active dense VS_2_ nanosheets were in‐situ deposited on the carbon cloth (as the cathode) and the Zn nanowires anode was obtained by electrodepositing on the surface of the carbon cloth by a three‐electrode system. Then, the integrated all‐in‐one flexible electrode in a battery was obtained for assembling the all‐in‐one wearable battery system.^[^ ^135^ ^]^ With the assistance of the PVA‐based hydrogel electrolyte, the flexible Zn//VS_2_ battery delivers the reversible self‐healing property. When the PVA‐based hydrogel was cut to a diameter of 5 cm and then connected for 30 min, two hydrogel pieces could tightly adhere and achieve a high loading of 500 g and keep well without obvious breaking. It could be ascribed to the good self‐healing property derived from the large amount of hydroxyl groups in the PVA skeleton. The self‐healing performance could also be improved by modulating the groups on the PVA polymer and the thickness of the hydrogel. Besides, the micro‐structure such as the pore diameter and the porous structure of the hydrogel also determinates the mechanical property and the self‐healing property, extending the practical application in portable and wearable electronic devices. Even after several seal‐healing processes by cutting vertically or horizontally, the battery could also light up the LED. As a result, the design of the all‐in‐one electrode or battery with the self‐healing property is desirable in the smart electronic devices of the future energy‐storage systems.

### 4.2 Self‐Protection Hydrogel Electrolytes

Safety is one of the most critical issues in the electrochemical energy storage devices. Dendrites are inevitably generated and growing during the repeated cycling process, which will finally lead to the internal short circuits and uncontrollable overcharging of batteries.^[^ ^146^, ^147^, ^148^ ^]^ In recent years, there are many reported explosions, fires, and other dangerous accidents of the electric vehicles due to these hazard factors.^[^ ^149^, ^150^ ^]^ In addition, a large amount of Joule heat during the cycling processes of the energy storage devices will be also the potential security risk. The kinetics reaction at high temperature and the limited heat‐removal system would also comprise the capacity or even is not working properly of the battery. The excessive heat accumulation in the narrow and small space of a working battery module would likely cause an explosion.^[^ ^151^ ^]^ Developing the battery with self‐protection functions is a promising approach to eliminating these safety hazards. To achieve the self‐protection function in working, the responsive materials should be introduced into the battery system. For example, functional hydrogels with various responsive abilities have been utilized the different scenarios to adapt the environmental change or the dangerous signals, including the biomedical field (the blood sugar monitoring) and the food packaging field (the moisture detection) in a timely manner. According to the above‐mentioned responsiveness characteristic, a series of hydrogels are fabricated and used in the aqueous to achieve the responsive property, such as the pH response and the temperature dependent hydrogel.^[^ ^38^, ^152^ ^]^ Herein, the recent advancement of the self‐protected ZIBs will be summarized and discussed in detail (**Table** **3**).

**Table 3:** Summary of the strategies and electrochemical performance of ZIBs based on the hydrogel electrolytes with self‐protection function.

Hydrogel electrolyte	Cathode	Capacity [mAh g^−1^]	Capacity retention	Self‐protection principle	Switch off time	Ref.
poly(2‐vinylpyridine) /ZnSO_4_	iodine/ Ndoped‐carbon	193.0 (0.14 A g^−1^)	85.0% after 250 cycles at 0.3 A g^−1^	pH‐responsive electrolyte based on de‐protonation of quaternary pyridinic groups	30 s	[38]
PNIPAM/AM/ Zn(CF_3_SO_3_)_2_	PANI	168.7 (0.1 A g^−1^)	/	thermal‐responsive hydrogel based on pore structure evolution	/	[152]
PAAm/ZnCl_2_	MnO_2_	≈370.0 (2 mA cm^−2^)	≈100.0% after 500 cycles at 10 mA cm^−2^	zinc chloride‐enriched hygroscopic hydrogel electrolyte	≈1 h	[153]
PNA/ZnSO_4_/ MnSO_4_	MnO_2_/ CNT	145.0 (0.1 A g^−1^)	≈100.0% after 500 cycles at 0.5 A g^−1^	Thermo‐responsive polymer based on sol‐gel transition	<15 s	[34]

#### 4.2.1 Overcharge Self‐Protection Hydrogel Electrolytes

The deep charging and deep discharging process of the battery will be inevitably at the cost of the decreased electrochemical performance such as the rated capacity and the cycling stability in the repeated charge/discharge process. In the traditional “rocking chair” lithium ion battery, the over‐discharged behavior would result in the external redox reaction of the copper ion derived from the conductive collector and finally diffuse into the liquid electrolyte. In contrast, the over‐charging process maybe cause the collapse of the positive cathode materials in the lithium ion de‐intercalation process. All of these results are not beneficial for achieving the long‐term cycling stability. In a working aqueous zinc battery, the external reaction in over‐charging or over‐discharging process will contribute to the decomposition reaction of water in aqueous electrolyte accompanied with the H_2_ and O_2_ evolution. It would increase the pressure of inside the working battery and induce the explosion to some extent in the limited space of the coin‐type battery or pouch battery. Therefore, it is desirable to detect the over‐charging behavior and effectively avoid the irreversible reaction. For example, Feng et al. have demonstrated a smart zinc‐iodine aqueous battery with an overcharge protection function.^[^ ^38^ ^]^ The primary smart material is the poly (2‐vinylpyridine) in the battery, which can complete the transition from the hydrophilic soluble state to the hydrophobic gel state within 30 s and then prevent the battery away from overcharging (Figure 8c). Herein, the pH of the electrolyte as the stimulus‐responsive inducement could effectively reflect the working situation of the working ZIB. When the zinc‐iodine battery was over‐charged, the internal resistance increases by four orders of magnitude because of the fast transformation of poly (2‐vinylpyridine) derived from the de‐protonation effect. The battery cannot normally work and rapidly switched off with the almost no capacity contribution. When the pH increases from 4 to 6, the resistance sharply increases from 10 to 220 ohms. It could effectively avoid the short circuit or even the fire in a working device system. Importantly, the self‐protected battery could also switch to the initial state accompanied with the recovery of the pH value in the battery system. As a result, the pH of the electrolyte was readjusted to the normal level by adding dilute sulfuric acid and the battery also restored to its normal capacity. The repeated overcharge/recovery cycle and the normal working capacity with almost no loss indicate that the smart self‐protection reversibility in a ZIB. This work opens a novel way for the development of overcharge‐protected ZIBs.

#### 4.2.2 Thermal Self‐Protection Hydrogel Electrolytes

Thermal self‐protection is an attractive strategy to achieve the operation safety of the battery especially at high temperature states and avoid the thermal runaway. The deionized water as the electrolyte solvent endows the diffusion of the positive and negative charges in the electrochemical process. The resistance variation in the electrolyte plays an important role in affecting the electrochemical performance of the Zinc battery. The resistance increases accompanied with the evaporation of water at high temperature and decreases the charge transfer (or ion diffusion), resulting in the sluggish reaction kinetic process or the poor operation of a working battery even shut down. When the temperature was recovered to the normal situation, the battery could also be normally charged and discharged for the energy conversion and storage. As the important component of the battery, the design of the hydrogel electrolyte with the thermal responsive ability is beneficial for achieving the thermal‐protection battery devices. For example, a smart hygroscopic hydrogel was fabricated for thermally self‐protected ZIBs based on the polyacrylamide (PAAm) hydrogel electrolyte.^[^ ^153^ ^]^ Herein, the ZnCl_2_ was chosen as the electrolyte salt because its saturated vapor pressure could be easily regulated by the concentration in the hydrogel electrolyte, which is an important factor in determining the thermal protection. When the battery was working at high temperature, the Zn‐PAAm with appropriate saturated vapor pressure evaporated water rapidly (Figure 8d). It causes the blocked zinc ion migration with an order of magnitude from 3.8 × 10^−10^ to 3.4 × 10^−11^ cm^2^ s^−1^ due to the evaporation of water and then the working battery was cut off. When the temperature recovered to room temperature again, Zn‐PAAm could absorb the water vapor in the air and resume normal work. When the battery was heated at 50.5 °C, the zinc ions could hardly migrate, and the capacity decreased rapidly because of the water evaporation and thermal resistance increase in aqueous ZIBs. When the temperature was re‐covered to the initial working state (normally the room temperature), the battery also delivers the matched capacity with almost no decay. This thermal reversibility also confirms the thermal self‐protection function based on the water content.

Although the porous structure is beneficial for the ion diffusion in the electrochemical process, they are not desirable at any temperature, especially at the high temperature. Niu et al. constructed the thermal self‐protection ZIBs employing thermal‐responsive porous poly(N‐isopropylacrylamide) (PNIPAM) hydrogel electrolytes.^[^ ^152^ ^]^ The PNIPAM formed intermolecular hydrogen bonds after exceeding its volume phase transition temperature (VPTT) and the PNIPAM molecular chain shrank and changed from hydrophilicity to hydrophobicity. To increase the VPTT from 33 to 45 °C, acrylamide incorporated with hydrophilic molecules was introduced into the PNIPAM hydrogel and obtained the PNIPAM/AM‐5 with the thermal self‐protection. When the temperature exceeded the VPTT, the exposed hydrophobic isopropyl of PNIPAM/AM‐5 changed from hydrophilicity to hydrophobicity accompanied with the disappearance of the porous structure and the ionic conductivity of the hydrogel electrolyte also decreased by about one order of magnitude. As a result, the PNIPAM/AM‐5 based ZIB displays the strong thermal response at high temperature and these changes were reversible when the temperature dropped to room temperature (Figure 8e). Even after several rapid temperature increases/decreases, the thermal self‐protection performance is not affected, and the battery could also achieve the good electrochemical performance.

It was worth noting that the smart thermal self‐protection function was not unique to PAAm or PAM hydrogels. The hydrogels with the sol‐gel transition point or the hydrophilic and hydrophobic functional groups play an important role in achieving the self‐protection property in the battery. For example, the PVA hydrogels are also utilized in battery to achieve the similar thermal self‐protection functions. When the temperature exceeded the sol‐gel transition point of the hydrogel, the polymer sol was transformed into the solid hydrogel, resulting in the poor ion migration in the electrochemical process. The strong temperature dependence of the hydrogel with the resistance change contributes to assembling the smart self‐protection ZIB. For example, a proton‐incorporated poly(N‐isopropylacrylamide‐co‐acrylic acid) (PNA) sol‐gel transition electrolyte was fabricated for the thermal self‐protection ZIBs.^[^ ^34^ ^]^ At the transition temperature, the hydrogen bonding force formed by PNA was stronger than that of the hydrophobic part because of the good water solubility of acrylic acid and PNA exists as the form of the transparent flowing sol. When the temperature increased to the phase transition point 50 °C, the hydrogen bond in the PNA sol was destroyed and the hydrophobic block structure mainly dominated the large part of the PNA chain (Figure 8f). It would affect the ion diffusion in the electrochemical process by decreasing the wettability of the electrolyte. The low transition time of 10 and 15 s exhibits the good reversibility. Even after multiple heating/cooling cycles, the battery still possesses the thermal self‐protection function, and the electrochemical performance had no obvious attenuation. The feasibility of the electrochemical performance based on the thermal self‐protection property provides the novel design of the hydrogel electrolyte, such as the molecular structure of the chain skeleton, the functional groups, and the hydrophobic and hydrophilic property for addressing the thermal runaway in the practical application in smart electronic devices.

### 4.3 Wide Operational Temperature Hydrogel Electrolytes

Aerospace, high altitude, deep sea and other special environments put forward higher requirements for the working temperature range within the battery.^[^ ^17^ ^]^ At the low‐temperature, the increase of electrolytes viscosity and the sluggish ion diffusion would decrease the ionic conductivity. At the same time, the low‐temperature environment could lead to the water icing and the separation of the electrode/electrolyte interface, which greatly hinders the ion migration on the electrode interface in a working battery. The electrochemical performance of battery is sharply reduced or even completely cut off at a low‐temperature. In contrast, when the battery was operated at high‐temperature conditions, the electrochemical capacity of the battery could increase at the initial state due to the accelerated ion diffusion. However, the moisture molecule in the electrolyte rapidly evaporates and the electrolyte salts accordingly precipitate with the increasing temperature, which greatly affects the long‐term cycling stability of the battery. In addition, the high temperature also affects the stability between the electrode and electrolyte such as the thermal instability of the electrode structure, which further deteriorate the electrochemical performance of battery.^[^ ^154^ ^]^ Therefore, the design of the battery that could operate at a wide temperature window is desirable for extending their practical application especially at extreme working fields.

#### 4.3.1 Decrease the Hydrogen Bond in the Aqueous Electrolytes

The abundant amount of water in the aqueous electrolyte would freeze and not normally work under the sub‐zero temperature situation, causing the poor ionic conductivity and large interface resistance according to the law of thermodynamics. These results decrease the electrochemical performance of the ZIB or even at the out‐of‐work state. To solve the frozen aqueous electrolyte at the low temperature, it is a significant factor to break the hydrogen bonds in the original aqueous electrolyte with abundant water molecules. A series of strategies have been developed to decrease the hydrogen bonds between the water molecules, such as the water‐soluble organic molecule additives in electrolyte, the antifreeze solvent, the high‐concentration electrolyte or hydrogel electrolyte. In addition, the aqueous electrolyte with only a little free water molecule is also desirable for achieving the weak hydrogen bonds and the good ionic conductivity and rational electrochemical performance. For example, an aqueous‐salt hydrates deep eutectic solvent of 3.5 M Mg(ClO_4_)_2_ and 1 M Zn(ClO_4_)_2_ was fabricated in ZIBs.^[^ ^155^ ^]^ The synergistic effect of anion and cation in the electrolyte destroyed the hydrogen bond among water molecules by altering the coordination environment of H atom and O atom, displaying an ultra‐low freezing point of −121 °C. Its good ionic conductivity and low viscosity are also ascribed to the pure water solvent, contributing to the rapid ion diffusion and low interface resistance in the charge/discharge process. Apart from the deep eutectic solvent, the modulation of the electrolyte structure by the ZnCl_2_ could also reduce the solid‐liquid transition point from 0 to −114 °C, and successfully realized the wide working temperature window from −90 to 60 °C.^[^ ^156^ ^]^ The solvation configurations of charge carrier (such as Zn^2+^) with the water molecules in the aqueous electrolyte plays an important role in maintaining the stability of the aqueous electrolyte and suppressing them to dissociate into free water. It is desirable for achieving the stable electrolyte at wide working temperature windows and the superior electrochemical performance in an aqueous battery system, especially extending their practical application in various electronic devices in future.

#### 4.3.2 The Organic Electrolyte or the Mixed Organic/Water Electrolyte

In addition to changing the electrolyte solute, the non‐aqueous organic solvent instead of the solvent water also achieved a wide operating temperature in ZIBs. A zinc trifluoromethanesulfonate electrolyte dissolved in N, N‐dimethylformamide was successfully developed and it could normally work at a wide temperature range from −70 to 150 °C. The large operation temperature range provides its potential application in extreme scenarios without the consideration of the low energy density in Zn‐organic battery.^[^ ^157^ ^]^ Although the regulation of the concentration of electrolyte salt and the organic electrolyte could broaden the working temperature of the zinc battery and provide the application at the low or high temperature states, the high cost of electrolytes with the water‐in‐salt type and the safety problem of organic problem still limit their wide application, which is a similar problem within the commercial lithium ion battery. It is noted that the weakened hydrogen bonds in aqueous electrolytes are desirable for achieving the wide operation temperature windows without the addition of the organic solvent or the high‐concentration electrolytes in the aqueous zinc battery. As a result, it is significant to decrease the amount of the active water content and increase the coordination interaction with the charge carrier (Zn^2+^) or the salt in the electrolyte, contributing to the higher or lower temperature operation and achieving the smart battery.

#### 4.3.3 The Development of the Quasi‐Solid Electrolytes

**Figure 9:**
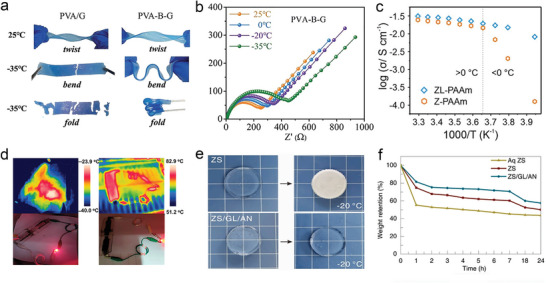
Wide operational temperature hydrogels in ZIBs. a) Photographs of PVA/G and PVA‐B‐G at 25 °C and ‐35 °C under twisting, bending, and folding states. b) Nyquist plots of PVA‐B‐G battery. a and b) Reproduced with permission.^[^ ^136^ ^]^ Copyright 2020, Royal Society of Chemistry. c) Variation in conductivity with and without LiCl. Reproduced with permission.^[^ ^137^ ^]^ Copyright 2019, Wiley‐VCH. d) Optical and infrared thermal imaging photos of the timer driven by the OHE‐based battery at ≈80 and −30 °C. Reproduced with permission.^[^ ^40^ ^]^ Copyright 2021, American Chemical Society. e) Photos of hydrogels at different temperatures. f) The weight retention of hydrogels at 60 °C. e and f) Reproduced with permission.^[^ ^138^ ^]^ Copyright 2022, Elsevier.

Low free water content in the quasi‐solid electrolyte also decreases the freezing point, commonly lower than the ice point. The rational design of the hydrogel electrolytes could also broaden the operation temperature window of the ZIBs. A large number of hydrophilic residues of the cross‐linked hydrogel polymer could connect the water molecules (bound water) in the network and decrease the content of the free water in the hydrogel. In general, free water is easy to form regular ice crystals and has a common freezing point (≈0 °C). Bound water could destroy the hydrogen bond among the free water and decrease the freezing point.^[^ ^131^ ^]^ Therefore, the lower free water content in the hydrogel leads to realize a lower ice point and contributes to the ion diffusion under 0 °C, which endows the normal electrochemical process of the battery. In addition, the hydrophobic residues in the polymer chain expand in contact with water and maintain the good mechanical property, which is also desirable for constructing the flexible smart devices at low‐temperature environment. Therefore, the hydrogel electrolytes with the limited existence of free water molecules provide a promising approach to realize wide operating temperature, especially at the low‐temperature operation situation of ZIBs.^[^ ^158^ ^]^ A borax‐crosslinked polyvinyl alcohol/glycerol gel electrolyte (PVA‐B‐G) with good flexibility has been developed to work at −35 °C (**Figure** **9**a).^[^ ^136^ ^]^ Glycerol as the water‐soluble solvent can not only destroy the strong hydrogen bond among free water molecules, but also react with the PVA chain and restrict the formation of the ice crystal in the hydrogel network, leading to a sharp decrease to −60 °C. In addition, with the synergistic effect of the Borax mediated the cross‐linking effect, the co‐crosslinking of glycerol and PVA greatly reduced the interaction among PVA chains and eliminated the formation of crystallization microdomains, which is also conducive to the charge transport. When the test temperature decreased from 25 to −35 °C, the charge transfer resistance only increased from 238 to 453 ohms (Figure 9b), which is much better than the increasing 62 times of the pristine state. Therefore, the design of the binary co‐solvent hydrogel could achieve a low freezing point, providing a low‐cost anti‐freezing gel electrolyte for flexible smart power supply.

#### 4.3.4 The Additives of the External Solutes in the Electrolytes

Without the additive of the external solvent, electrolyte solutes with a highly hydrated synergistic cationic are efficient for decreasing the content of free water and achieving the anti‐freezing hydrogel electrolyte. For example, the sodium chloride salts could keep the road from freezing on rainy or snowy days under subzero temperatures. The anti‐freezing tolerance of the salts is derived from the ion hydration of concentrated solutions with many ionic compounds, which can decrease the temperature of the ice crystallization in water. Based on the above‐mentioned results, the inorganic salts with small ionic radii and weak coordination of metal ions such as alkali metal salt could weaken the intermolecular hydrogen bonds and extend the working temperature range. For example, apart from the strong hydration ions SO_4_^2−^ and Zn^2+^, the high hydration number of Li^+^ could also significantly weaken the intermolecular hydrogen bonding.^[^ ^137^ ^]^ When the temperature is below 0 °C, the ionic conductivity of polyacrylamide hydrogel with LiCl salt decreased slightly due to the auxiliary hydration of Li^+^ (Figure 9c). Typical discharge curves were also observed after resting at −20 °C for 24 h. The development of the cooperative hydrated cations is a promising approach to obtain the anti‐freezing hydrogel with good flexible property in achieving the high‐performance zinc battery over a wide working temperature range. The hydrogel electrolyte with its low cost and high safety is desirable for assembling the flexible smart devices in extreme conditions.

#### 4.3.5 The Multifunctional Electrolytes for the Integrated Devices

Modulating the structure of electrolyte solvents such as the mixed solvent of the electrolytes is also an important way to realize wide operating temperature ZIBs. The regular formation of ice crystals in water solution is ascribed to the strong hydrogen bonds among free water molecules. When the external soluble organic solvent was added to the aqueous solution, the initial hydrogel bond would be destroyed, and the corresponding ice crystal cannot generate. As a result, the freezing point would decrease to some extent according to the formula of freezing point reduction in the colligative properties of dilute solutions. For example, a binary ethylene glycol (EG)/H_2_O solvent hydrogel electrolyte with ZnCl_2_/NH_4_Cl salts was utilized to realize the ZIB operating from −30 to 80 °C.^[^ ^40^ ^]^ When the hydrogel electrolyte was operated at a high temperature above 40 °C, its high ionic conductivity is attributed to the high evaporation of water in the binary EG/H_2_O solvent, which is much higher than only H_2_O solvent. The good ionic conductivity also affects the electrochemical performance with a wide operating temperature range (worked for more than 60 days at −20 °C) (Figure 9d). Even the temperature is 80 °C, the battery could also normally operate for more than 1000 times. Apart from the good ionic conductivity at low temperature, the normal charge/discharge process at high temperature also determinates its wide application. In addition, soluble organic solvents with hydrophilic groups such as ‐OH, ‐COOH, ‐NH_2_ could serve as the active sites for adsorbing the water molecule, which weakens the strong interaction among water molecules. It could be beneficial for decreasing the content of free water in an aqueous solution and extending the operating temperature range. As proof of the concept, an anti‐freezing/thermally stable hydrogel electrolyte (ZS/GL/AN) composed of polyacrylamide (PAM), glycerin (EG), acetonitrile (AN), and zinc sulfate was developed.^[^ ^138^ ^]^ The high electron density of oxygen and ammonium in PAM and GL could regulate the Zn^2+^ shell structure and destroy the hydrogen bonds among water molecules. Water molecules that form hydrogen bonds with PAM or GL were considered as bound water, which was locked in the hydrogel 3D interpenetrating network. The existence of bound water would destroy the lattice of water molecules and reduce the activity of water by weakening the internal hydrogen bond, which endows the hydrogel electrolyte with wide temperature range stability and successfully realizes the normal operation of ZIBs from −20–60 °C. The integrated hydrogel electrolyte possesses the lower polarization and longer cycle performance (more than 500 h) at low‐temperature or high‐temperature in comparison with ≈50 h in traditional liquid electrolytes. Benefiting from the strong affinity between water and electron‐rich oxygen groups and the large amount of moisture holding in the 3D hydrogel networks, the formation of ice crystals at low temperature and the evaporation of water molecules at high temperature is effectively limited (Figure 9e,f). It provides a novel strategy for realizing the normal operation of ZIBs at a wide temperature window and extends its application in the deep space and deep sea. Apart the above‐mentioned strategies to achieve the wide operation temperature, the hydrogel based on the ionic liquid is also the promising candidate for fabricating the smart device at a high working situation due to its high ionic conductivity, non‐evaporation, and thermal stability.^[^ ^159^ ^]^

In the development of ZIB with a wide working temperature range, the water content in hydrogels is always inevitably discussed. The lattice structure of water molecules is arranged neatly, which leads to their easy freezing at low‐temperature, complete closure of ion migration channels, and decline or even complete loss the batteries performance. The addition of some electron cloud‐dense groups such as ‐OH, ‐COOH, ‐NH_2_ can destroy hydrogen bonds among water molecules and reduce the freezing point of water molecules. For high‐temperature, due to the high saturated vapor pressure of water, the rapid evaporation of water molecules in the hydrogel leads to a sharp increase in salt concentration and even salt precipitation, which seriously hinders the normal migration of Zn^2+^. It is very effective to adjust the saturated vapor pressure of electrolytes by adding some different salts. Tightly locking the water molecules into hydrogels is the primary problem for the realization of wide operating temperature ZIBs.

## 5 The Smart ZIB and the Integrated Application Fields

### 5.1 The Design Principles for Smart ZIBs Components

**Figure 10:**
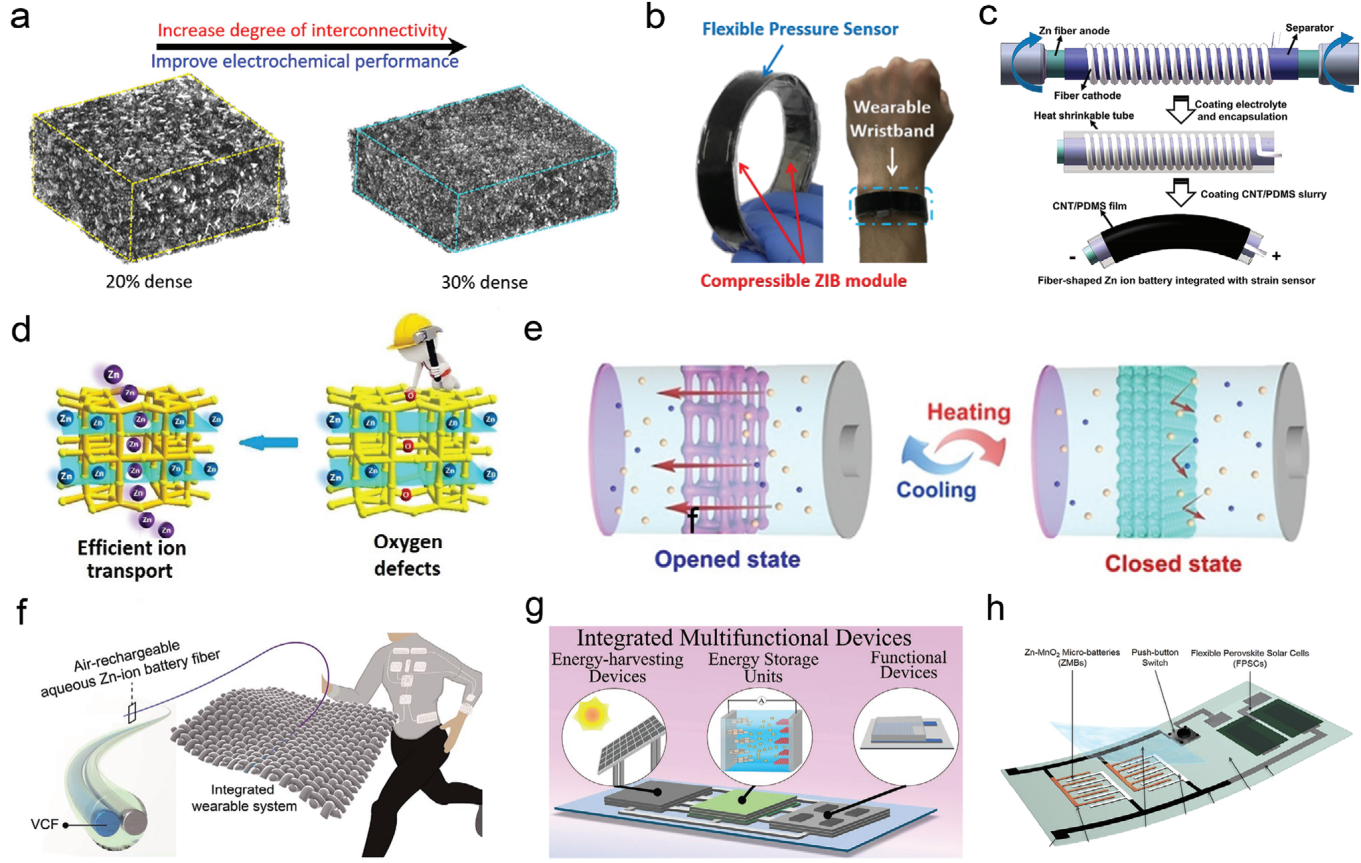
The design principles of smart ZIBs and their integrated application fields. a) 3D volume‐rendered X‐ray tomogram of zinc sponges of different densities. Reproduced with permission.^[^ ^201^ ^]^ Copyright 2019, American Chemical Society. b) Flexible smart wristband integrated from two ZIB modules and a flexible pressure sensor. Reproduced with permission.^[^ ^196^ ^]^ Copyright 2018, American Chemical Society. c) Demonstration of the preparation process of the multifunctional battery fiber. Reproduced with permission.^[^ ^194^ ^]^ Copyright 2022, American Chemical Society. d) Schematic images of the defective crystal with efficient ion transports. Reproduced with permission.^[^ ^202^ ^]^ Copyright 2020, Elsevier. e) Schematic illustration of smart ZIBs with thermal‐gated PNIPAM/AM electrolytes. Reproduced with permission.^[^ ^152^ ^]^ Copyright 2020, Wiley‐VCH. f) Schematic of the aqueous ZIB fiber with air‐recharging capability integrated into multifunctional wearable systems. Reproduced with permission.^[^ ^44^ ^]^ Copyright 2021, Royal Society of Chemistry. g) Device design and principle of the integrated multifunctional devices strategy.^[^ ^197^ ^]^ Reproduced with permission. Copyright 2022, Wiley‐VCH. h) Schematics of the device configuration of the integrated flexible photo‐rechargeable system. Reproduced with permission.^[^ ^203^ ^]^ Copyright 2022, Elsevier.

For the active zinc anode, the adaptability and stability of the zinc anode is necessary for achieving the high performance and prolonging the cycling life.^[^ ^160^ ^]^ The inherent ductility and flexibility of the metal zinc, zinc foil, zinc fiber, zinc rod are widely common and utilized in the smart ZIB configuration. The contents of active zinc sites for the reversible deposition and precipitation and the corresponding ductility is a significant role in the assembly of the smart devices. The alloying mixed zinc metal is beneficial for uniform deposition of active zinc ion on the mixed zinc surface by providing abundant zinc nucleation sites for zinc deposition in charge/discharge process. For example, the zincophilic noble metals such as Ag or Au are introduced into the zinc metal and serve as the nucleation sites for inducing the uniform distribution of the metal zinc ion. In addition, the conductive substrate as the zinc host possesses the large surface area and the interworking conductive network structure is also beneficial for the flexible battery, such as the fabrication of the porous zinc, zinc sponge, zinc layer (**Figure** **10**a).^[^ ^161^, ^162^ ^]^ The large specific surface area enables the uniform charge distribution and contributes to the oriented deposition on the conductive framework such as the Cu substrate, graphene, porous activated carbon. The formation of the compact zinc particle layer could further effectively suppress the formation of zinc dendrites. Besides, the mixed zinc anode could increase the nucleation barrier and limit the 2D diffusion of Zn^2+^ and promote the uniform deposition of Zn^2+^. The mixed zinc anode could avoid the direct contact with the aqueous electrolyte, which greatly decreases the hydrogen gas production during the electrochemical process, and reduces the side effects on the bare zinc metal anode. The mixed zinc anode can not only endow the battery with the reversible redox reaction process, but also decrease the side reaction on the metal zinc surface in the electrochemical process.

For the cathode materials, the additional functionality of cathode materials is considered to simplify the configuration in the smart integrated battery devices apart from the normal zinc ion storage.^[^ ^163^ ^]^ It requires the development of the cathode composites through the various physical recombination or chemical synthesis. For example, the selection of cathode materials with the functional integration of the air‐charging, the photo‐sensitive, or the self‐charging property is also taken into consideration apart from the zinc ion storage. The content of the inactive material in the cathode is reduced as much as possible on the basis of meeting these intelligences integrations.^[^ ^164^ ^]^ In addition, the corresponding electrochemical performance of the cathode materials and the energy density of the smart devices cannot be at the cost of the functional integration in the devices because of the cathode as the energy carrier. Therefore, the balance of the capacity, cycling life, and the energy density of the smart battery is the cardinal principle.

For the electrolytes in smart integrated devices, the quasi‐solid or solid state electrolytes with the low free‐water content play a key role in improving the mechanical performance of intelligent integrated devices, especially in the flexible devices at special working condition or some extreme situation (self‐protection, self‐healing, and environmental compatibility).^[^ ^165^ ^]^ For the integrated battery, the selection of the polymer chain, functional groups, and the hydrophilic property as well as the water contents can interact with Zn^2+^ through the electrostatic interaction to regulate Zn^2+^ deposition. Moreover, the hierarchical porous frameworks of hydrogels also provide the uniform, stable, and rapid transfer pathway for electrolyte ions. The outstanding mechanical properties and adhesive effects of hydrogel materials could effectively restrict growth of the zinc dendrites on the interface of the metal zinc anode, but also contribute to the realization of flexible and integrated devices in the intelligentization process of ZIBs. The external additives in the electrolytes are also desirable for achieving the stimulus‐responsive hydrogel electrolytes of the smart battery devices. Besides, the oxygen content, the moisture content, as well as the air atmosphere in the electrolytes are also the critically influencing factors in affecting electrochemical performance and the cycling life in repeated charge/discharge process of the smart ZIB as well as their utilization fields.

For the assembly structure of the smart integrated devices, the simple assembly method, stable energy output and collection, and the high integration of various functional units are the three important designing principles in the smart ZIBs.^[^ ^166^ ^]^ Planar‐type battery and the flexible battery are the common energy supplying in the smart device structure and they are applicable to the various application scenarios according to the different requirements (Figure 10b). 1) As the representative of the planar device, the sandwich‐type battery is a simple preparation approach through the layer‐by‐layer stacking model.^[^ ^167^ ^]^ Among which, the interfacial contact is the main challenge for realizing the fast charge transfer and the ion diffusion in the electrochemical process. The good interfacial contact contributes to the reversibly chemical redox reaction and promotes the utilization of the active materials for the high performance and good cycling stability. In addition, the interdigital electrode structure of the battery is desirable to promise the integration of the micro‐battery in the portable devices or the small‐area chip and realize the large‐area functional integration in the smart zinc battery. 2) For the flexible electronic devices, the development of the fibrous and the soft pack batteries is necessary due to its good mechanical stability and compatibility under the external forces such as the different bending or folding states (Figure 10c).^[^ ^168^ ^]^ Moreover, their large volumetric density, good stretchability, and wearability are beneficial for the wearable electronics with the display function, pressure sensing, or the energy harvesting property.

### 5.2 The Self‐Powered Battery with the Energy Harvesting

The green and sustainable energy supply in electronic devices is a prerequisite to ensure the charging/discharging process at the normal state and special state of the equipment.^[^ ^169^, ^170^, ^171^ ^]^ Self‐charging via the air or the solar is the common strategy to achieve the self‐powered devices.^[^ ^172^, ^173^, ^174^, ^175^, ^176^, ^177^ ^]^ For the efficient utilization of a single self‐charging device type in consideration of the energy saving field, the property of self‐charging in active electrode is attractive in aqueous ZIB, which also determinates the electrochemical performance of the working battery. Therefore, based on the afore‐mentioned self‐charging mechanism, the rational design of the active electrodes is conducive to the energy harvesting for promoting the self‐powered battery system. 1) It is desirable for improving the electrochemical performance by optimizing the micro‐morphology and the electronic structure of the cathode materials. As shown in Figure 10d, the design of the controlled defects in the vanadium oxides could contribute to the reversible utilization in the charge/discharge process, especially at a low current density.^[^ ^178^, ^179^, ^180^ ^]^ The optimization of the active cathode materials plays an important role in achieving the self‐charging battery system and maintaining the good performance in the long‐term cycle process. 2) To decrease the manufacturing cost and the external energy consumption, the multifunctional cathode materials will also play an important role in the complex energy supply system. For example, the energy storage device is integrated with the smart electrochromic characteristic via a simple approach, extending the application of the energy supply field.^[[^ ^181^, ^182^ ^]^ The response of the color change at different voltage plateaus could serve as a detector for the real‐time capacity display, which delivers the good response to the external environment changes. It is also a significant response parameter to detect the energy harvesting and supply in a working electronic device.^[^ ^96^, ^183^ ^]^ To obtain the smart ZIB with the sustainable energy harvesting property is also attractive in practical applications and the corresponding design of the active electrodes or the smart packages also promotes the production landing in future.

### 5.3 The Environmental Self‐Adaptation Battery with the Flexibility

The environmental self‐adaptions refer to the ability of the battery to achieve all the intended performance and function without being destroyed under the effect of the comprehensive environmental factors at a working state (such as at the state of the energy supply in the discharging process).^[^ ^184^, ^185^, ^186^ ^]^ With the development of the wearable electronic devices such as the smart electronic skin, the wearable electronic display clothing, or the flexible electronic equipment terminal, the corresponding self‐adaption energy supply (battery system) is also derived.^[^ ^187^, ^188^, ^189^, ^190^ ^]^ The external changes including the temperature, force, or the light would determinates the electrochemical performance. The concrete embodiment of the battery's ability is to adapt to the environment.

The flexible and wearable battery with high security and stability could extend their practical application. 1) The design of the smart electrodes is based on the environmental response. The timely self‐regulation under the changes of the external environment is in great demand of the smart battery. For example, the high temperature working situation usually appears in summer or some special area. Based on the thermal response requirement, the smart ZIB with the functional electrolyte has been fabricated.^[^ ^191^ ^]^ Within the rational temperature working window, the battery could provide the theoretical capacity. When the external surrounding exceeds the temperature window, the capacity of battery decreases, or the battery is not working with the failure (Figure 10e). The concept is also widely utilized in the design of the battery module. However, the separate detector and the battery could not achieve the fast response at the critical situation, which would result in a lot of financial damage. Therefore, the smart battery with the response ability is desirable in future. 2) The assembly of the wearable battery for adapting the external force change. The bulk and rigid coin‐type battery in mobile phones or battery modules in the electric car is common in our daylily life.^[^ ^192^ ^]^ However, the poor mechanical deformation is not suitable for the wearable electronic or smart clothes. The hydrogel electrolyte as the flexible carrier with low water content, relative ionic conductivity, and the reversibly chemical interface between electrode and electrolyte has attracted increasing attention, especially for the wearable devices with good flexibility (Figure 10f). Based on the hydrogel electrolyte, the assembled battery could normally work under the suitable mechanical strength such as the bending, twisting, or even folded states. In addition, the certain adhesion of the hydrogel electrolyte with flexibility also contributes to promoting the integrating degree, avoiding the active electrode separation or battery failure derived from the external force. Therefore, to extend the practical application in the wearable electronics with the environmental self‐adaptation, the smart battery based on the functional hydrogel electrolyte is the key factor. The self‐adaptation property of the hydrogel should possess the good ionic conductivity, crush resistance, anti‐high and low temperature as well as the high water conservation.

### 5.4 The Integrated System Based on Smart Zinc Battery

As the energy supply system, the next generation of the intelligent electronic devices plays a significant role in energy storage and conversion, which also presents demands for the convenient energy supply methods and highly integrated device structures. The widely reported integration systems include the energy supplying and energy harvesting fields in the smart ZIB. However, the traditional device integration is on the basis of the indirect connection with the external power supply.^[^ ^130^, ^164^ ^]^ The discontinuous external power grid supply is an inconvenient and complicated process for realizing the stability of the integrated intelligent battery system.^[^ ^193^ ^]^ This phenomenon will be exacerbated in the emerging micro‐integrated systems and the wearable devices. The simple assembly method, the stable energy output and collection, and the high integration of various functional units are the three important designing principles in the smart ZIB. Based on the above‐mentioned designing principles, the assembled smart battery will contribute to achieving the convenience, compatibility, and efficiency during the energy supplying process and further improving the user experience.

1. As the energy supplying system, the integration of the smart ZIBs with various sensor parts effectively solve the discontinuous supply of external power.^[^ ^194^, ^195^ ^]^ More importantly, the integrated self‐power sensors could also avoid the short comings of the multi‐device integration, such as the multi‐step process, compatibility and interface stability. The high‐efficiency energy supplying is also desirable for the multi‐functional electronic devices.^[^ ^196^ ^]^ The integration of the ZIB and sensor unit within a single device possesses both sensing and energy storage and supplying is a new strategy for realizing next‐generation miniature integrated devices with superior miniaturization, flexibility and durability (Figure 10g). To achieve the miniaturization and high integration of electronic devices, the following challenges and solutions of the integrated smart ZIB should be also taken into consideration.^[^ ^197^ ^]^ a) The electrochemical stability and compatibility as well as the cost of the micro‐battery are necessary for the long‐term cycling life. b) The Young's modulus is an important parameter to evaluate the commercialization of the flexible ZIBs. c) The toxicity, degradability and renewable value of the integrated devices (sensor) are also considered.
2. As the energy harvesting system, the integration of the smart ZIB with the photo‐rechargeable system (solar battery) or the friction power generation system (triboelectric nanogenerator) could realize the reversible energy storage and conversion.^[^ ^198^, ^199^ ^]^ The effective integration could significantly reduce the inner volume space and the weight of the wearable electronics, which is also beneficial for the utilization and portability.^[^ ^197^ ^]^ It is also the direct strategy to achieve the collection of discontinuous resources, and provide the continuous energy supplying in the integrated system for electronic devices in future. The connection between the ZIB and the solar cells is activated by the simple push‐button switch within the integrated system. The collected luminous energy is utilized to charge the ZIB and obtain the passive self‐charging device (Figure 10h). In addition, the electrostatic energy generated by the friction could also be converted into the electric energy, which provides a green and sustainable power supply. The combination of micro‐battery and the mechanical energy (such as the triboelectric nanogenerator) serves as the main body of the energy supply and promises the normal working of the microelectronic device. However, there are several issues to be solved in the integrated energy harvesting systems in the practical application. a) The matching of voltage and current of integrated devices determines the energy density and service life in energy harvesting systems. b) The recycling and secondary utilization of the smart ZIB and the mechanical devices in the integrated system are also taken into consideration, especially for the large‐scale application of the wearable electronic devices. c) The new packaging technology such as the 3D printing approach also plays an important role in solving the interfacial contact and the fabrication and design of the microstructure electrode.
3. The intelligent management based on the smart battery. The release of heat from battery module maybe result in the failure of the battery supply. Moreover, the large heat release in a short lead to the explosion in a limited space. Therefore, the good sensitivity of the smart battery with thermal response could avoid or decrease the insecurity derived from the thermal runaway.^[^ ^200^ ^]^ Apart from the thermal response, the force response or the photo response of the smart battery is also desirable in the detection system of intelligent devices in future.

## 6 Conclusion and Perspectives

The development of the smart ZIBs as a new type of intelligent energy storage device has attracted great attention on the road to the high‐security and low‐cost as well as the self‐adapting battery system. In this review, the design of the cathode and anode materials and the development of the corresponding hydrogel electrolytes in the aqueous ZIB are summarized in detail. To further extend the practical application of the integrated functional aqueous battery and avoid the challenges in fabricating the active electrodes and the smart interface among the electrode interfaces and battery system, the potential strategies and the corresponding perspectives are in the following:

### 6.1 The Development of Smart Active Electrode Materials

The design of the active cathode and anode electrodes plays an important role in charging/discharging process for the development of the smart ZIBs. For example, when the smart materials serve as active electrodes for achieving the self‐charging or electrochromic device, the capacity degradation of the electrode derived from the leakage and evaporation of the electrolytes in an open battery system and the color shift caused by the degradation of the active cathodes would accelerate the degradation of the electrode modules during the long‐term operation and leads to the functional failure of the smart devices based on the ZIBs. In addition, the relatively low specific capacity and the operation voltage of the smart electrode still limit the energy storage capability and cycling stability for realizing the practical application. There are several strategies to fabricate the stable and high‐performance electrodes in the intelligentization process of ZIBs.

For cathode materials, a) the doping of metal ions or heteroatoms (N, P, S, O atoms) of cathode materials could improve the energy density and rate performance of the ZIBs by enhancing the stability and conductivity of the cathode crystalline structure. b) The rational design of the interlayer spacing and morphology of the cathode materials. It cannot only accelerate the kinetic redox reaction, but also provide the external active sites for anchoring much more zinc ion or anion by introducing the vacancy defects, which contributes to improving the electrochemical performance. c) The chemical activity and reversibility of the cathode with a high working voltage. It is also necessary for achieving the high‐energy density, such as the dual‐ions intercalation and de‐intercalation redox mechanism or other multi‐ions insertion reaction (ion capacitor combined with the battery) in the cathode materials.

For smart zinc anode materials, a) the adjustment of the chemical composition (such as zinc alloying) and structure of the zinc anode is beneficial in providing nucleation sites for zinc ions and achieving uniform charge distribution, thereby suppressing the formation of zinc dendrites. b) The functional surface coating on the zinc anode effectively physically isolates the electrolyte from the surface of the zinc and forms the uniform interfacial protective layer, thereby mitigating the occurrence of hydrogen evolution side reactions and the dendrite growth for realizing the good cycling stability and high energy density. Furthermore, the corresponding smart package of the battery is also desirable for prolonging the cycling stability. Therefore, the development of smart active electrode materials is dependent on the rational regulation of the surface properties, the chemical reversibility, the electronic structure, the micro‐morphology, and the assembled strategy of cathodes and anodes as well as the smart package are necessary for achieving the high energy storage capability and cycling stability.

### 6.2 The Development of the Smart Response Hydrogel Electrolytes

Hydrogel electrolytes as an important component integrate the dual functions of separator and electrolyte because of their relatively high ionic conductivity and excellent electrode compatibility in ZIBs. In addition, the excellent flexibility of hydrogel electrolytes also provides an important way to realize wearable electronic devices in the age of intelligence. In this review, for the design of the smart hydrogel electrolytes, the working principle and the research progress of self‐healing, self‐protection and wide working temperature range hydrogels are discussed in detail. The ZIBs based on the smart hydrogel electrolyte could timely self‐regulate under the changes of the external environment. However, the efficiency and stability of the self‐healing and self‐protection functions of current hydrogel electrolytes are insufficient, which cannot provide the applicability in practical application. In addition, most of the smart functions of hydrogels are synergistic, which also limits the targeted response requirement in a working battery. As a result, to extend the utilization of the smart hydrogel electrolyte, the development of hydrogel electrolytes with multiple smart functions and the chemical reversibility is the significant factor in guaranteeing the stability of ZIBs and the corresponding adaptability under the complex working environments. a) The relatively high ionic conductivity. Electrolyte as the charge diffusion carrier determinates the electrochemical performance. The rapid charge transfer in the charge/discharge process is desirable, especially at the fast‐charging field. b) The appropriate flexibility. The design of the flexible hydrogel electrolyte could effectively avoid the leakage of the electrolyte and the induced short circuit and the environmental problem in the traditional aqueous working battery. Moreover, the wearable electronic devices are usually in direct contact with the human skin, and the development of flexible hydrogels for wearable electronic devices should be harmless to the human body and other substrate material. From the perspective of the ecological environment, the degradable and biocompatible hydrogel electrolytes also have great potential for alleviating the environmental pollution. c) The targeted specificity. The reversible ability to recover the functional hydrogel is also desirable for extending the application under the extreme conditions (such as the anti‐fatigue ability, the high elasticity, and the ultra‐low temperature).

### 6.3 The Development of the Integrated Smart Device

Smart electronic devices are desirable in the development of the miniaturization and multi‐function to meet the needs of the portable and diversified applications. Therefore, the smart ZIB with the integrated systems could provide energy storage and energy conversion, but also possess the super functional units such as the self‐charging, and self‐healing property. The integrated system also plays an important role in delivering the energy supplying even under the condition of tolerating a variety of complex environmental changes. The superior performance is ascribed to the integrated high compatibility and durability requirements among functional integration units in integrated electronic devices. The temperature adaptability also requires the wide temperature range of the aqueous electrolyte in the smart integrated ZIB. For the wearable electronic devices, the electrochemical stability under the bending states and the cycling performance determinate the application scenarios of the wearable electronic devices. The wearable electronic devices based on the smart ZIB are suitable for diverse application scenarios such as wearable electronic devices, electronic skins and flexible textiles in comparison with the traditional rigid substrates. Importantly, the non‐toxic and low harmless characteristics are necessary for achieving the comfort and safety in the utilization of the electronic devices. The smart ZIBs could eliminate concerns about device compatibility and durability by simplifying the complex integrated device configurations. Aqueous ZIB as a green and sustainable energy supply via the reversible chemical redox reaction can not only promote the optimization of energy structure, but also contribute to the low carbon transition. Therefore, the new‐type integrated system is also in great need for improving the energy storage and conversion, such as the construction of the aqueous battery mixed with the sensors, the energy storage derived from the solar battery, as well as the integration with the traditional devices. There are also some challenges in the integration process between the different types of devices. a) The interface compatibility of the electrode materials in all‐in‐one devices. b) The reversible stability during the electrochemical process. c) The lifespan of the integrated smart devices. All the above‐mentioned issues should be taken into consideration, especially in a complex working process. Therefore, the application of the computer simulation (such as the artificial intelligence technology or the ChatGPT) will provide effective strategies for smart devices.

As the component of the smart response devices, the selection and design of the active electrode will also induce the unsatisfactory electrochemical performance of a working zinc battery due to the sacrifice the ionic conductivity and the working voltage window in the electrochemical process. As a result, the reported ZIBs with smart responses only stay at the concept stage and cannot achieve the widespread application. It is noted that a satisfactory balance should be achieved between the inherent electrochemical performance sacrifice and the introduction of smart functions in an integrated device. In addition, the smart properties lifespan of the smart ZIBs is far shorter than the lifespan of the traditional ZIBs. It is also desirable for developing the reversible and stable active materials for prolonging the lifespan. Consequently, the successful and initial achievement of the smart ZIBs device in the laboratory stage will provide the commercialization of large‐scale industrial production in future.

## Conflict of Interest

The authors declare no conflict of interest.
